# One-pot double annulations to confer diastereoselective spirooxindolepyrrolothiazoles

**DOI:** 10.3762/bjoc.18.171

**Published:** 2022-11-28

**Authors:** Juan Lu, Bin Yao, Desheng Zhan, Zhuo Sun, Yun Ji, Xiaofeng Zhang

**Affiliations:** 1 Department of Chemistry, Changchun Normal University, Changchun 130031, P. R. China; 2 Department of Civil Engineering, University of North Dakota, 243 Centennial Drive Stop 8115, Grand Forks, North Dakota 58202, United States; 3 Department of Chemical Engineering, University of North Dakota, 241 Centennial Drive Stop 7101, Grand Forks, North Dakota 58202, United States; 4 Department of Cancer Biology, Dana-Farber Cancer Institute, Harvard University, Boston, MA 02215, USA; 5 Broad Institute of Harvard and MIT, Cambridge, MA 02142, United States

**Keywords:** azomethine ylides, cascade, double annulations, *N,S*-acetalation, pyrrolothiazoles, spirooxindole

## Abstract

A novel four-component reaction in one pot as an atom- and step-economic process was developed to synthesize diastereoselectively spirooxindolepyrrolothiazoles through sequential *N,S*-acetalation of aldehydes with cysteine and decarboxylative [3 + 2] cycloaddition with olefinic oxindoles. High synthetic efficiency, operational simplification and reaction process economy using EtOH as solvent, and only releasing CO_2_ and H_2_O as side products confer this approach favorable in green chemistry metrics analysis.

## Introduction

Nitrogen-containing heterocycles play a dominant role as a structural fragment of therapeutic agents in medicinal chemistry and drug discovery [1–9]. The nitrogen-containing heterocyclic moieties are currently discovered in more than 75% of the drugs available in the market approved by the FDA. Thus, the reaction process with synthetic efficiency and operational simplification is a critical factor in the construction of nitrogen-based heterocycles. Normally, some advantageous approaches in green synthesis are in favor of innovating the synthetic methods, optimizing the reaction process and eliminating the step of intermediate purification to save resources and reduce waste [10–12]. The pot, atom, step and economic (PASE) approach [13–17] is one of the most distinguished representatives in the efficient synthesis of nitrogen-based heterocycles, such as multicomponent reactions (MCRs) [18–23], one-pot cascade reactions [24–32] as good examples of PASE synthesis. We have reported a series of multicomponent reactions, like Groebke–Blackburn–Bienayme for making BET inhibitors UMB32 and UMB136 [33–34]. Zhang developed 4-aminoquinolines for the synthesis of fluorinated analogues of acetylcholinesterase (AChE) inhibitors [35] in cascade reactions, such as one-step syntheses of quinolines. Quinolin-4-ols involving histone acetyltransferases (HAT) inhibitors [36–37], as well as one-pot reactions were also developed by Zhang using the 4-aminoquinoline synthesis, for example, in amino acids(esters)-based [3 + 2] cycloadditions [38–48] and in the synthesis of pyrrolidine-containing systems [49–59]. Pyrrolothiazole and spirooxindole moieties occupy exclusive positions as valuable source of natural products and therapeutic agents in organic synthesis and drug discovery [60–68].

**Scheme 1:**
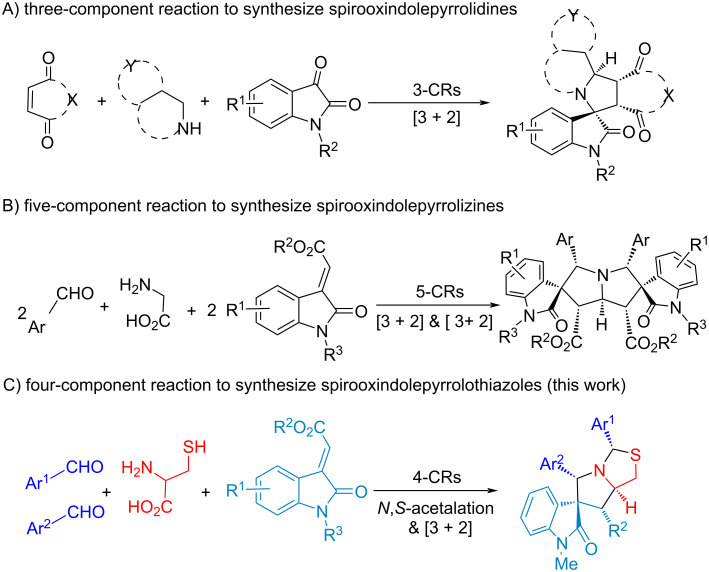
The diastereoselective synthesis of spirooxindoles through MCRs.

**Figure 1:**
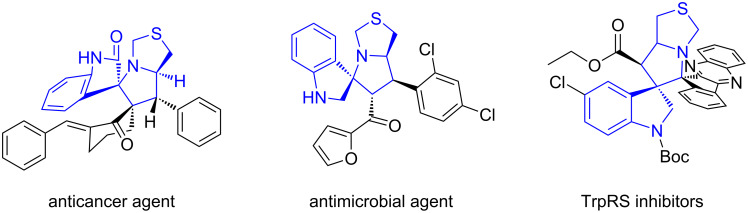
Bioactive Spirooxindole-pyrrolothiazoles.

**Scheme 2:**
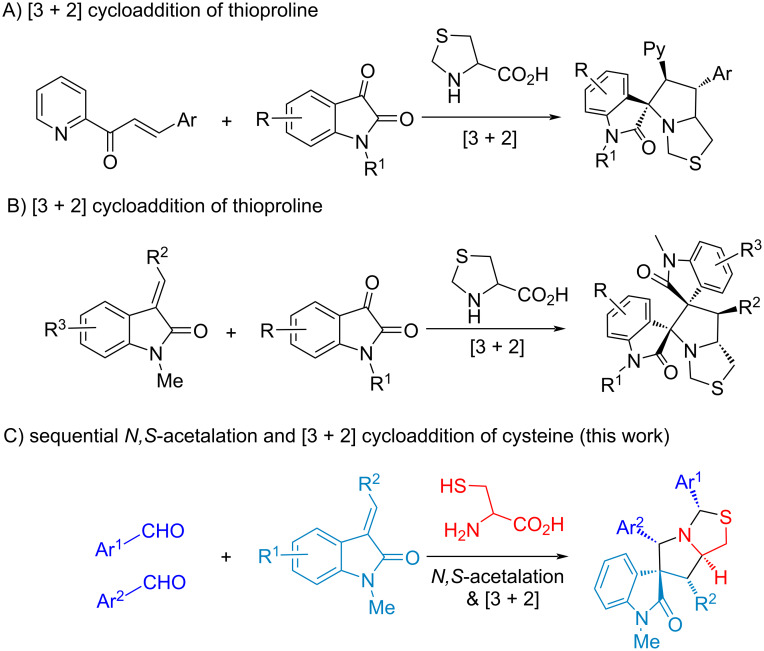
The synthesis of spirooxindolepyrrolothiazoles.

We have developed a number of asymmetric reactions to construct spirooxindole-based scaffolds through one-pot reactions with recyclable organocatalysts [69]. Notably, we conferred K10 acid to promote the C–H activation in the synthesis of spirooxindolepyrrolidines, and used Zeolite HY catalyst to synthesize diastereoselective dispiro[oxindolepyrrolidine]s with a butterfly shape (Scheme 1A and 1B) [70–71]. With the promising applications of spirooxindolepyrrolothiazoles in drug discovery (Figure 1) [72–74], the structural integration of spirooxindole and pyrrolothiazole with diverse substituted groups via an efficient synthesis is a challengeable research in green chemistry. The corresponding PASE reactions of making spirooxindolepyrrolothiazoles are even more rare, which only involves three-component reactions with isatins and thioproline (Scheme 2A and 2B) [75–76].

Four-component double annulations through 2-substituted thioprolines formed in *N,S*-acetalation of aldehyde and cysteine was introduced in this study. Subsequently one equivalent of aldehyde and olefinic oxindole in situ were followed by decarboxylative 1,3-dipolar cycloaddition for diastereoselective synthesis of spirooxindolepyrrolothiazoles with generating 5 new bonds, 5 stereocenters and two heterocycles (Scheme 1C and Scheme 2C).

## Results and Discussion

The optimized reaction conditions of stepwise, one-pot and cascade (two-step with one operational step) processes for *N,S*-acetalation and decarboxylative 1,3-dipolar cycloaddition were developed by using two equivalents of 4-bromobenzaldehyde (**1a**), ʟ-cysteine (**2**) and olefinic oxindole **4a** shown in Table 1. In our continuous effort on the reaction optimization of benign solvents, we firstly evaluated the influence of reaction time and protic solvents such as EtOH, iPrOH and MeOH at 25 °C for 6 h, which only results in slightly different LC yield (93–95%) of compound **3a** (Table 1, entries 2–4) superior to 86% yield for 3 h (Table 1 entry 1), and followed by decarboxylative [3 + 2] cycloaddition with the second equivalent of compound **1a** and olefinic oxindole **4a** under reflux heating for 12 h. It indicates that the one-pot reaction process with EtOH and iPrOH afforded the 81% of LC yield for compound **5a** slightly better than 78% yield using MeOH as a solvent (Table 1, entries 2–4). After screening the reaction temperature in the 2nd step of the one-pot process (Table 1, entries 4 and 5), it was found that the diastereomeric mixture of thioproline **3a** without purification from *N,S*-acetalation with 1.0:1.15 of **1a**/**2** at 25 °C for 6 h with EtOH as solvent, in situ followed by addition of 1.1:1.0 of **1a**/**4a** for [3 + 2] cycloaddition at 90 °C for 9 h gave compound **5a** with the 81% of LC yield. Next, the stepwise process was also carried out by using the thioproline **3a** (1 equiv) with 86% of isolated yield and 1.1:1.0 of **1a**/**4a** through decarboxylative [3 + 2] cycloaddition (Table 1, entry 6), which afforded compound **5a** with 73% isolated yield at 90 °C for 9 h. Notably, we conferred cascade reaction process to synthesize compound **5a** with 70% isolated yield as one-step four-component reaction (4-CR) with 2.2:1.1:1.0 of **1a**/**2**/**4a** at 90 °C for 9 h in EtOH after variations of solvents, reaction time and temperature with one operational step (Table 1, entries 7–12). This 4-CR is the first example of double annulations with sequential *N,S*-acetalation and [3 + 2] cycloaddition for diastereoselective spirooxindolepyrrolothiazoles by the formation of two new rings, 5 bonds, and 5 stereocenters without intermediate purification.

**Table 1:** Optimization of reaction conditions for double annulations of cysteine.^a^

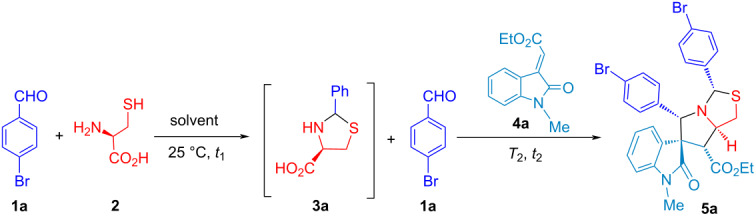						

entry	solvent	*t*_1_ (h)	**3a** (%)^b^	*t*_2_ (h)	*T*_1_(°C)	**5a** (%)^b^

1	EtOH	3	86			–
2	iPrOH	6	95	12	105	81
3	MeOH	6	93	12	70	78
4	EtOH	6	95	12	90	81
5	EtOH	6	95	9	90	81
6^c,e^	EtOH	6	95 (86)	9	90	83 (73)
7^d,e^	EtOH			9	90	79 (70)
8 ^d^	EtOH			6	90	67
9 ^d^	EtOH			18	90	75
10^d^	MeOH			9	70	76
11^d^	iPrOH			9	105	78
12^d^	MeCN			9	90	67

^a^One-pot reaction of 1.0:1.15 of **1a**/**2** for *N*,*S*-acetalation **3a** followed by addition of 1.1:1.0 of **1a**/**4a** for [3 + 2] cycloaddition.^b^Detected by LC–MS, isolated yield in parenthesis. ^c^Intermediate **3a** was isolated in the two-step reaction. ^d^Cascade reaction of 2.2:1.1:1.0 of **1a**/**2**/**4a**. ^e^dr > 4:1, Determined by ^1^H NMR analysis of the crude products after the reaction mixture filtered through a pad of silica gel and removal of solvent.

**Scheme 3:**
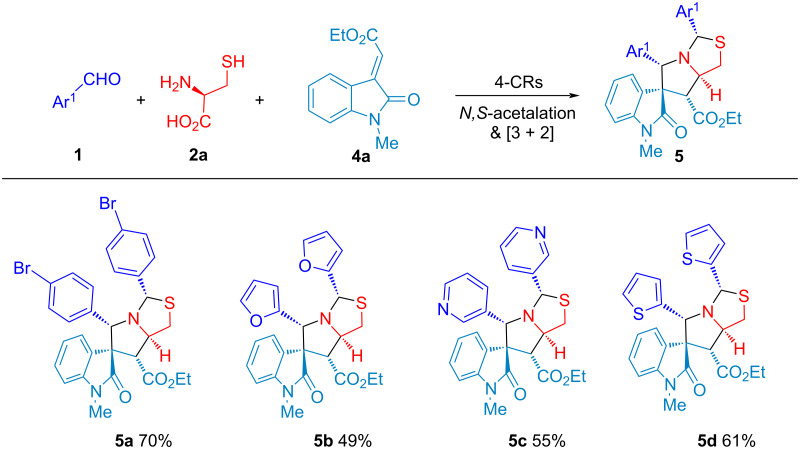
Four-component reaction for the synthesis of compound **5**.

To explore the reaction scope of 4-CR, different aldehydes **1** (Ar^1^) were used to react with ʟ-cysteine (**2**) and olefinic oxindole **4a** in the synthesis of substituted spirooxindolepyrrolothiazole analogues **5a**–**d** with 49–70% isolated yield (Scheme 3) under the optimized reaction conditions (Table 1, entry 7). Compounds **5b**–**d** with using heteroaromatic aldehydes resulted in lower yield than **5a**.

In addition, according to the one-pot reaction process (Table 1, entry 5) with two operational steps using different aldehydes **1** and **6**, products **7a**–**e** were synthesized in 43–72% isolated yields and up to 6:1 dr (Table 2).

**Table 2:** One-pot reaction for the synthesis of compound **7**.

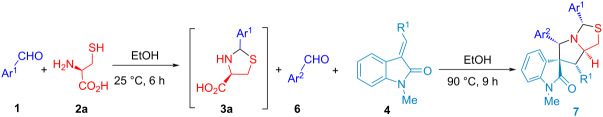					

entry	Ar^1^	Ar^2^	R^1^	product	yield (%)^b^

1	2-thiophenyl	3-OMe-4-FC_6_H_3_	CO_2_Et	**7a**	66
2	2-thiophenyl	2-furanyl	CO_2_Et	**7b**	51
3	2-thiophenyl	3-pyridinyl	CO_2_Et	**7c**	43
4	2-FC_6_H_4_	4-ClC_6_H_4_	CO_2_Et	**7d**	72
5	4-BrC_6_H_4_	4-ClC_6_H_4_	CO_2_Et	**7e**	66
6	4-BrC_6_H_4_	Ph	COMe	**7f**	trace
7	4-BrC_6_H_4_	Ph	Ph	**7g**	–
8	4-BrC_6_H_4_	CO_2_Et	CO_2_Et	**7h**	messy
9	4-BrC_6_H_4_	ethyl	CO_2_Et	**7i**	messy

*^a^*Isolated yield. Reaction conditions are same as Table 1, entry 5.

**Scheme 4:**
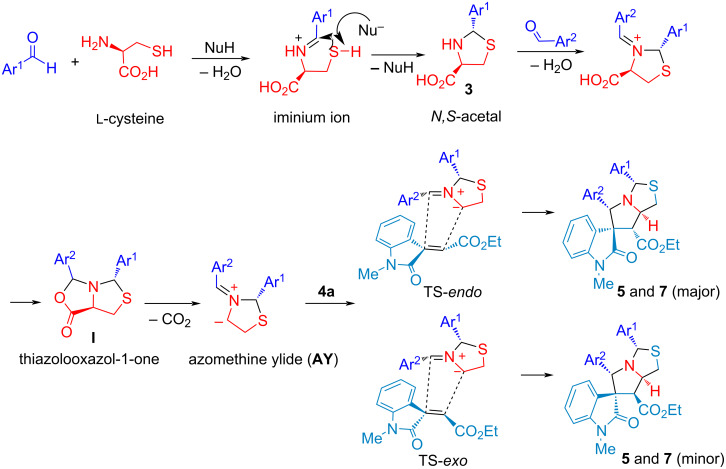
Proposed mechanism for the double [3 + 2] cycloadditions.

The results indicate that the substituent on Ar^2^ of the aldehydes could influence the product yield, such as **7c** (3-pyridinyl, 43% yield, 4.5:1 dr). In addition, oxindole **4** with different R^1^ was employed for the synthesis to give **7f** with COMe in a trace amount and no product **7g** with a Ph group. The following reactions with aliphatic aldehydes gave **7h** and **7i** as complex mixtures [54–59,71]. The reaction mechanism of the double annulations for sequential *N,S*-acetalation and decarboxylative [3 + 2] cycloaddition is shown in Scheme 4. With the promotion of the protonic solvent EtOH, compound **3** (*N,S*-acetal) from the condensation of cysteine and an aldehyde reacts with a second equivalent of aldehyde followed by cyclization to generate thiazolooxazol-1-one **I**.

**Scheme 5:**
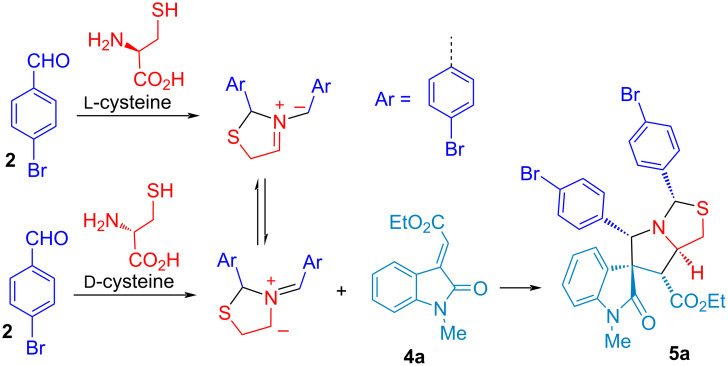
The synthesis of compound **5a** with ᴅ- and ʟ-cysteine.

**Scheme 6:**
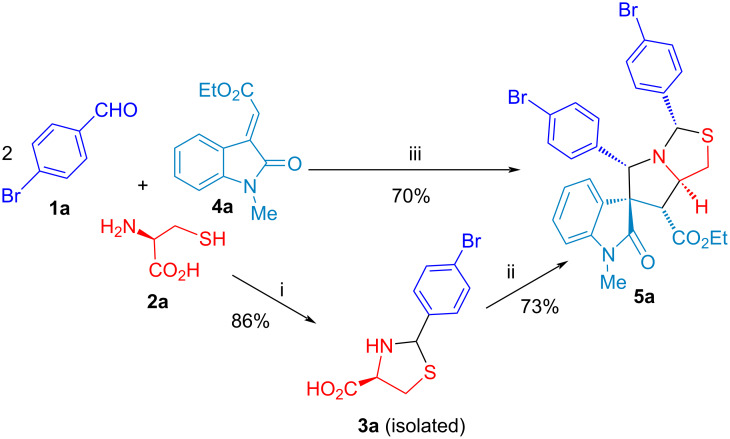
Two-step (process A) vs cascade (process B) synthesis of **5a**. i) 1.0:1.15 of **1a**/**2**, EtOH (0.05 M), 25 °C, 6 h. ii) 1.1:1.0:1.0 of **1a**/**3a**/**4a**, EtOH (0.5 M), 90 °C, 9 h (Table 1, entry 6). iii) 2.2:1.1:1.0 of **1a**/**2**/**4a**, EtOH (0.5 M), 90 °C, 9 h (Table 1, entry 7).

**Figure 2:**
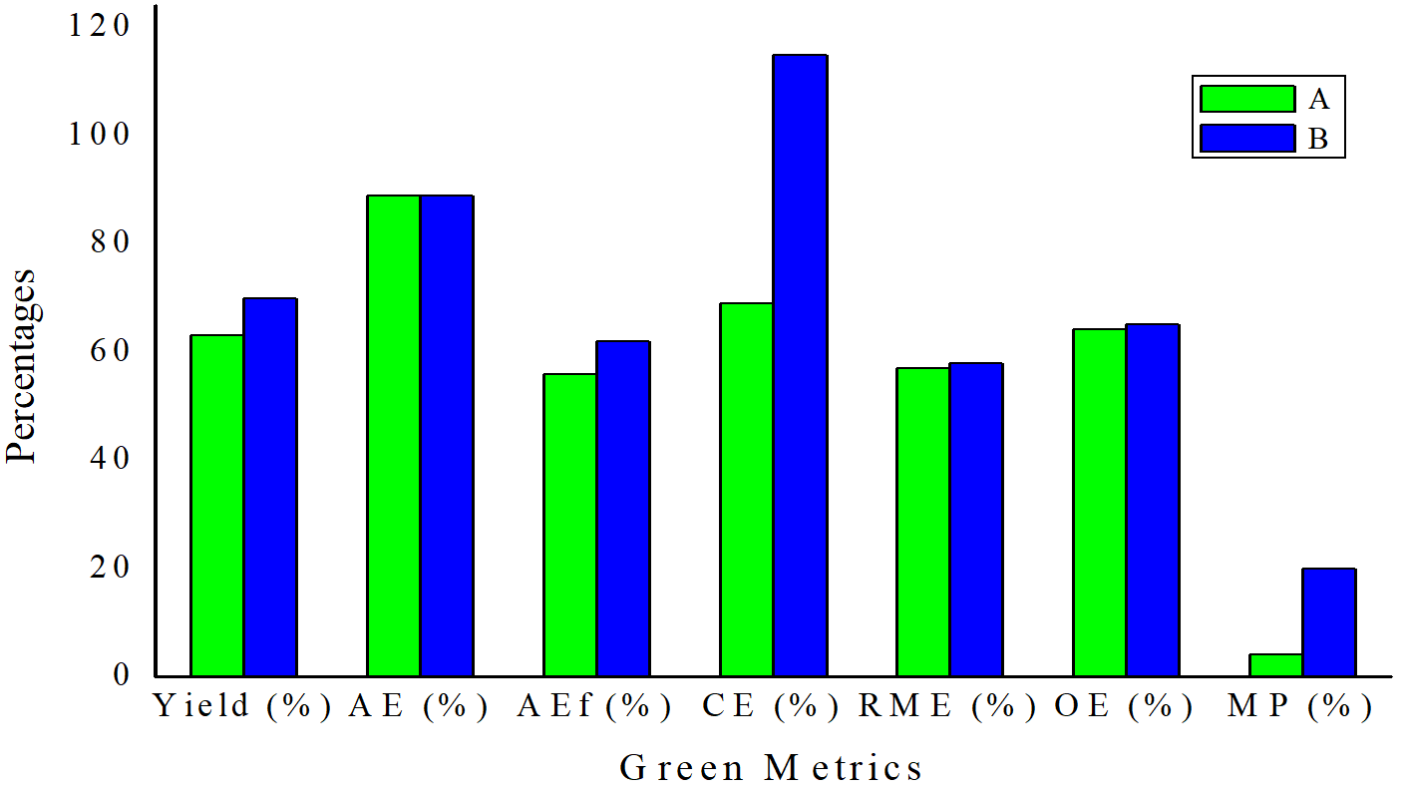
Graphical representation of the green metrics (AE, AEf, CE, RME, OE and MP) analysis for processes A and B. The higher the value, the greener the process.

**Figure 3:**
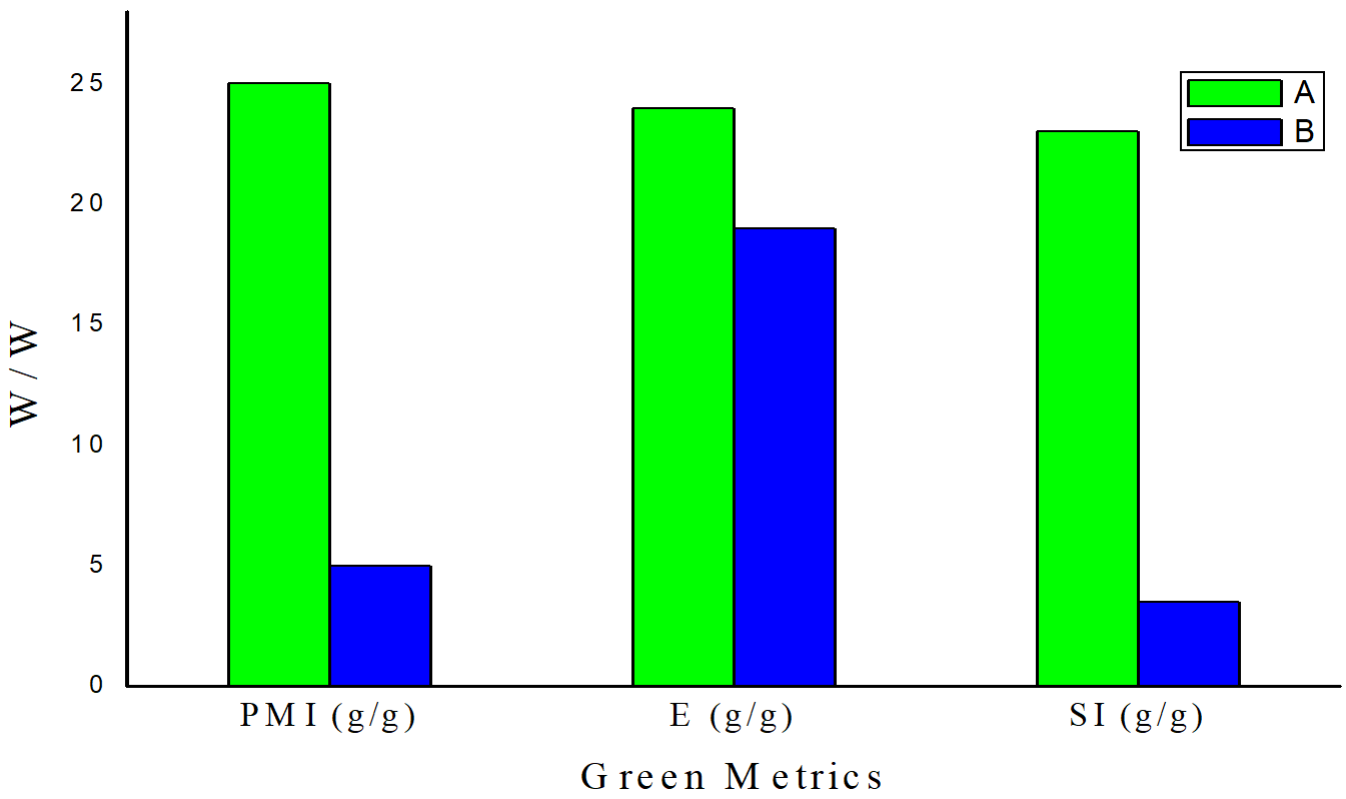
Graphical representation of the green metrics (PMI, E-factor, and SI) analysis for processes A and B. The lower value, the better the reaction process.

Subsequent decarboxylation of thiazolooxazol-1-one **I** affords non-stabilized azomethine ylide (**AY**) for 1,3-dipolar cycloaddition with olefinic oxindole **4a** to give spirooxindolepyrrolothiazoles **5** and **7**. The *endo*-TS is more favorable than *exo*-TS for the 1,3-dipolar cycloaddition to afford the major and minor products. The diastereochemistry of non-stabilized azomethine ylides for decarboxylative [3 + 2] cycloaddition could be identified in reported literature [54–59,71]. Through the study of the mechanism, it elucidates that the double annulations using ʟ-cysteine undergoes three stages: compound **3**, thiazolooxazol-1-one **I** and **AY** in the reducing stereocenter in an ratio of 3 to 1. The mechanistic process indicates that the configuration of ʟ-cysteine didn’t affect the stereoselectivity in the formation of compound **5** and **7**. Thus, we further validated the hypothesis through the experimental results using ᴅ- and ʟ-cysteine to synthesize compound **5a** (Scheme 5). We conferred green chemistry metrics to evaluate the process efficiency of four-component reaction via comprehensive and quantitative calculation. The metrics analysis is carried out for the two-step synthesis with intermediate separation (process A) and the single-step method (process B) for the synthesis of spirooxindolepyrrolothiazoles **5a** according to the reaction conditions shown in Scheme 6. Green chemistry metrics data including atom economy (AE), atom efficiency (AEf), carbon efficiency (CE), reaction mass efficiency (RME), optimum efficiency (OE), mass productivity (MP), mass intensity (MI), process mass intensity (PMI), E factor (E), and solvent intensity (SI) are listed in Table 3 and Table 4 and are shown in Figure 2 and Figure 3 (the green metrics and detailed calculation process is described in Supporting Information File 1).

**Table 3:** Green metrics (AE, AEf, CE, RME, OE and MP) analysis for processes A and B.

process	isolation steps	yield (%)	AE (%)	AEf (%)	CE (%)	RME (%)	OE (%)	MP (%)

A	2	63	88.9	56	64.4	57	64.1	4
B	1	70	88.9	62	115	58	65	20

**Table 4:** Green metrics (PMI, E-factor, and SI) analysis for processes A and B.

process	PMI (g/g)	E (g/g)	SI (g/g)

A	25	24	23
B	5	19	3.5

Process A is a two-step method involving isolation of intermediates, in which compound **3a** was purified before 1,3-dipolar cycloaddition. Process B is a single-step approach without isolation of intermediate **3a**. The same substrates for synthesizing product **5a** in processes A and B results in 88.9% of AE. The AEf, RME and OE for one-step process B are 62%, 58% and 65%, a little better than those for process A (56%, 57% and 64.1%). In addition, CE and MP are significant references to elucidate reaction process consumption. The CE and MP for process B (115% and 20%) are much better than that for process A (64.4% and 4%). PMI for process A (25) is 5 times larger than that for process B (5). The E-factor for process A (24) is less significant than that for process B (19). Solvent consumption (SI, 3.5) for process B is clearly lower than that for process A (23) with more solvent for intermediate separations.

## Conclusion

A readily and efficient four-component synthesis for spirooxindolepyrrolothiazoles is introduced, which involves a sequential *N,S*-acetalation and decarboxylative [3 + 2] cycloaddition reaction. This one-pot and two-step process with four components generates 5 bonds, 5 stereocenters and two heterocycles in a diastereoselective fashion, and without intermediate purification. The one-pot four-component synthesis in green metrics analysis is compared with the stepwise reaction process to pinpoint the overwhelming advantages of the one-pot approach in the CE, MP, PMI, and SI by eliminating the intermediate purification. It is an efficient way to build up novel spirooxindolepyrrolothiazoles for drug discovery screening.

## Supporting Information

File 1 Experimental and analytical data, copies of NMR spectra, green metrics and the detailed calculation process.

## References

[1] Grover G, Nath R, Bhatia R, Akhtar M J (2020). Bioorg Med Chem.

[2] Kerru N, Gummidi L, Maddila S, Gangu K K, Jonnalagadda S B (2020). Molecules.

[3] Lang D K, Kaur R, Arora R, Saini B, Arora S (2020). Anti-Cancer Agents Med Chem.

[4] Heravi M M, Zadsirjan V (2020). RSC Adv.

[5] Rodriguez del Rey F O, Floreancig P E (2021). Org Lett.

[6] Deiters A, Martin S F (2004). Chem Rev.

[7] Shan Y, Su L, Zhao Z, Chen D (2021). Adv Synth Catal.

[8] Hemmerling F, Hahn F (2016). Beilstein J Org Chem.

[9] Kaur N (2019). Synth Commun.

[10] Clarke P A, Santos S, Martin W H C (2007). Green Chem.

[11] Trost B M (2002). Acc Chem Res.

[12] Anastas P, Eghbali N (2010). Chem Soc Rev.

[13] Zhang X, Zhang W (2018). Curr Opin Green Sustainable Chem.

[14] Newhouse T, Baran P S, Hoffmann R W (2009). Chem Soc Rev.

[15] Bhuyan D, Sarma R, Dommaraju Y, Prajapati D (2014). Green Chem.

[16] Hayashi Y, Umemiya S (2013). Angew Chem, Int Ed.

[17] Zhang W, Yi W-B, Zhang W, Yi W-B (2019). Introduction to PASE Synthesis. Pot, Atom, and Step Economy (PASE) Synthesis.

[18] Cioc R C, Ruijter E, Orru R V A (2014). Green Chem.

[19] Rotstein B H, Zaretsky S, Rai V, Yudin A K (2014). Chem Rev.

[20] Estévez V, Villacampa M, Menéndez J C (2014). Chem Soc Rev.

[21] de Graaff C, Ruijter E, Orru R V A (2012). Chem Soc Rev.

[22] Dömling A, Wang W, Wang K (2012). Chem Rev.

[23] Brauch S, van Berkel S S, Westermann B (2013). Chem Soc Rev.

[24] Tietze L F, Brasche G, Gericke K M (2006). Domino Reactions in Organic Synthesis.

[25] Nicolaou K C, Chen J S (2009). Chem Soc Rev.

[26] Enders D, Grondal C, Hüttl M R M (2007). Angew Chem, Int Ed.

[27] Padwa A, Bur S K (2007). Tetrahedron.

[28] Nicolaou K C, Edmonds D J, Bulger P G (2006). Angew Chem, Int Ed.

[29] Wasilke J-C, Obrey S J, Baker R T, Bazan G C (2005). Chem Rev.

[30] Hayashi Y (2016). Chem Sci.

[31] Sydnes M O (2014). Curr Green Chem.

[32] Atkinson M B J, Oyola-Reynoso S, Luna R E, Bwambok D K, Thuo M M (2015). RSC Adv.

[33] McKeown M R, Shaw D L, Fu H, Liu S, Xu X, Marineau J J, Huang Y, Zhang X, Buckley D L, Kadam A (2014). J Med Chem.

[34] Huang H, Liu S, Jean M, Simpson S, Huang H, Merkley M, Hayashi T, Kong W, Rodríguez-Sánchez I, Zhang X (2017). Front Microbiol.

[35] Zhang X, Ma X, Qiu W, Awad J, Evans J, Zhang W (2020). Adv Synth Catal.

[36] Zhang X, Dhawan G, Muthengi A, Liu S, Wang W, Legris M, Zhang W (2017). Green Chem.

[37] Zhang X, Ma X, Qiu W, Evans J, Zhang W (2019). Green Chem.

[38] Padwa A, Pearson W H (2003). Synthetic Applications of 1,3-Dipolar Cycloaddition Chemistry Toward Heterocycles and Natural Products.

[39] Coldham I, Hufton R (2005). Chem Rev.

[40] Pandey G, Banerjee P, Gadre S R (2006). Chem Rev.

[41] Hashimoto T, Maruoka K (2015). Chem Rev.

[42] Gothelf K V, Jørgensen K A (1998). Chem Rev.

[43] Martina K, Tagliapietra S, Veselov V V, Cravotto G (2019). Front Chem (Lausanne, Switz).

[44] Zhang W (2013). Chem Lett.

[45] Narayan R, Potowski M, Jia Z-J, Antonchick A P, Waldmann H (2014). Acc Chem Res.

[46] Selva V, Selva E, Merino P, Nájera C, Sansano J M (2018). Org Lett.

[47] Henke B R, Kouklis A J, Heathcock C H (1992). J Org Chem.

[48] Yildirim O, Grigalunas M, Brieger L, Strohmann C, Antonchick A P, Waldmann H (2021). Angew Chem, Int Ed.

[49] Lu Q, Song G, Jasinski J P, Keeley A C, Zhang W (2012). Green Chem.

[50] Zhang W, Lu Y, Geib S (2005). Org Lett.

[51] Zhang X, Qiu W, Ma X, Evans J, Kaur M, Jasinski J P, Zhang W (2018). J Org Chem.

[52] Zhang X, Zhi S, Wang W, Liu S, Jasinski J P, Zhang W (2016). Green Chem.

[53] Zhang X, Pham K, Liu S, Legris M, Muthengi A, Jasinski J P, Zhang W (2016). Beilstein J Org Chem.

[54] Zhang X, Liu M, Zhang W, Legris M, Zhang W (2017). J Fluorine Chem.

[55] Zhang X, Qiu W, Evans J, Kaur M, Jasinski J P, Zhang W (2019). Org Lett.

[56] Ma X, Zhang X, Qiu W, Zhang W, Wan B, Evans J, Zhang W (2019). Molecules.

[57] Ma X, Zhang X, Awad J M, Xie G, Qiu W, Muriph R E, Zhang W (2020). Tetrahedron Lett.

[58] Ma X, Meng S, Zhang X, Zhang Q, Yan S, Zhang Y, Zhang W (2020). Beilstein J Org Chem.

[59] Ma X, Qiu W, Liu L, Zhang X, Awad J, Evans J, Zhang W (2021). Green Synth Catal.

[60] Spanò V, Barreca M, Cilibrasi V, Genovese M, Renda M, Montalbano A, Galietta L J V, Barraja P (2021). Molecules.

[61] Noda K, Terasawa N, Murata M (2016). Food Funct.

[62] Noda K, Yamada S, Murata M (2015). Biosci, Biotechnol, Biochem.

[63] Bharkavi C, Vivek Kumar S, Ashraf Ali M, Osman H, Muthusubramanian S, Perumal S (2016). Bioorg Med Chem.

[64] Arulananda Babu S, Padmavathi R, Ahmad Aslam N, Rajkumar V, Atta-ur-Rahman (2015). Recent Developments on the Synthesis and Applications of Natural Products-Inspired Spirooxindole Frameworks. Studies in Natural Products Chemistry.

[65] Zhou L-M, Qu R-Y, Yang G-F (2020). Expert Opin Drug Discovery.

[66] Panda S S, Jones R A, Bachawala P, Mohapatra P P (2017). Mini-Rev Med Chem.

[67] Wang Y, Cobo A A, Franz A K (2021). Org Chem Front.

[68] Ye N, Chen H, Wold E A, Shi P-Y, Zhou J (2016). ACS Infect Dis.

[69] Huang X, Zhang W (2021). Chem Commun.

[70] Zhang X, Liu M, Qiu W, Evans J, Kaur M, Jasinski J P, Zhang W (2018). ACS Sustainable Chem Eng.

[71] Zhang X, Qiu W, Murray S A, Zhan D, Evans J, Jasinski J P, Wang X, Zhang W (2021). J Org Chem.

[72] Lotfy G, Said M M, El Ashry E S H, El Tamany E S H, Al-Dhfyan A, Abdel Aziz Y M, Barakat A (2017). Bioorg Med Chem.

[73] Wu G, Ouyang L, Liu J, Zeng S, Huang W, Han B, Wu F, He G, Xiang M (2013). Mol Diversity.

[74] Ren W, Zhao Q, Yu M, Guo L, Chang H, Jiang X, Luo Y, Huang W, He G (2020). Mol Diversity.

[75] Li J, Wang J, Xu Z, Zhu S (2014). ACS Comb Sci.

[76] Feng T-T, Gong Y, Wei Q-D, Wang G-L, Liu H-H, Tian M-Y, Liu X-L, Chen Z-Y, Zhou Y (2018). J Heterocycl Chem.

