# Supplementary material for: One-pot double annulations to confer diastereoselective spirooxindolepyrrolothiazoles
Source: Beilstein J Org Chem. 2022 Nov 28;18:1607–16. doi: 10.3762/bjoc.18.171 (PMC9727273; doi:10.3762/bjoc.18.171)
Supplement: File 1 — Experimental and analytical data, copies of NMR spectra, green metrics and the detailed calculation process. [file Beilstein_J_Org_Chem-18-1607-s001.pdf]

BEILSTEIN JOURNAL OF ORGANIC CHEMISTRY

Supporting Information

for

One-pot double annulations to confer diastereoselective spirooxindolepyrrolothiazoles

Juan Lu, Bin Yao, Desheng Zhan, Zhuo Sun, Yun Ji and Xiaofeng Zhang

Beilstein J. Org. Chem. **2022,** *18,* 1607–1616. doi:10.3762/bjoc.18.171

Experimental and analytical data, copies of NMR spectra, green metrics and the detailed calculation process

License and Terms: This is a supporting information file under the terms of the Creative Commons Attribution License (https://creativecommons.org/licenses/by/4.0). Please note that the reuse, redistribution and reproduction in particular requires that the author(s) and source are credited and that individual graphics may be subject to special legal provisions.
The license is subject to the *Beilstein Journal of Organic Chemistry* terms and conditions: (https://www.beilstein-journals.org/bjoc/terms)

Content list

1. General information……………………………………………………………..….S2
2. General procedures ……………………………………………………………… S2
3. Characterization of products………………………………………………………S3
4. NMR spectra of products …………………………………………………………S6
5. Green chemistry metrics analysis…..……………………………………………..S15
6. References ………………….………………………………………………….………S20

1. General information

All solvents were used as received from commercial sources without further purification. ^{1}H NMR and ^{13}C NMR spectra were recorded using Bruker-DRX (400 MHz and 101 MHz, respectively) instruments internally referenced to SiMe4, chloroform, and dimethyl sulfoxide signals. Chemical shifts were reported in parts per million (ppm), Multiplicity was indicated as follows: s (singlet), d (doublet), t (triplet), q (quartet), m (multiplet), dd (doublet of doublet), br s (broad singlet). LC-MS were performed on Waters UPLC-MS. The mobile phases were MeCN and H_2O both containing 0.03% HCO_2H. UV detections were conducted at 220 nm, 254 nm and 284 nm. Low resolution mass spectra were recorded in APCI (atmospheric pressure chemical ionization).

2. General procedure

2.1 The cascade synthesis of compound 5

To a solution of aldehydes **1** (2.2 mmol) and cysteine **2** (1.1 mmol), in 2.0 mL of EtOH was added olefinic oxindoles **4** (1.0 mmol). After being stirred at 90 °C for 9 h. Upon the completion of the reaction as monitored by LCMS, the concentrated reaction mixture was isolated. The products **5** were afforded.

2.2 The one-pot synthesis of compound 7

To a solution of aldehydes **1** (1.0 mmol) and cysteine **2** (1.15 mmol), in 3.0 mL of EtOH was added, then stirred at 25°C for 6 h. olefinic oxindoles **4** (1.0 mmol) and aldehydes **6** (1.1 mmol) were added. The solution mixture was stirred at 90 °C for 9 h. Upon the completion of the reaction as monitored by LCMS, the concentrated reaction mixture was isolated. The products **7** were afforded.

3. Characterization of products

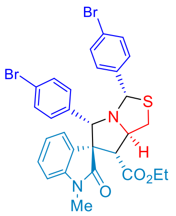

Compound ***5a:*** white solid (70% yield). ^{1}H NMR (400 MHz, $CDCl_3$) δ 7.60 – 7.56 (m, 1H), 7.46 (d, J = 8.5 Hz, 2H), 7.39 – 7.34 (m, 2H), 7.15 (td, J = 7.7, 1.2 Hz, 1H), 7.08 (d, J = 8.5 Hz, 2H), 7.03 (dd, J = 7.5, 1.0 Hz, 1H), 6.99 – 6.95 (m, 2H), 6.61 – 6.56 (m, 1H), 5.16 (s, 1H), 4.83 – 4.77 (m, 1H), 4.72 (s, 1H), 3.82 (dq, J = 10.8, 7.1 Hz, 1H), 3.68 – 3.59 (m, 2H), 3.16 – 3.09 (m, 4H), 3.00 (dd, J = 12.3, 1.7 Hz, 1H), 0.68 (t, J = 7.1 Hz, 3H).^{13}C NMR (101 MHz, $CDCl_3$) δ174.8, 169.1, 143.7, 140.8, 135.3, 131.3, 133.0, 128.9, 128.9, 128.2, 126.0, 125.2, 122.3, 121.8, 121.2, 107.9, 75.3, 74.7, 67.5, 61.3, 60.7, 56.2, 38.8, 26.4, 13.5. HRMS (ESI-TOF*, m/z*): $[M+H]^+$ calcd. for $C_{29}H_{26}Br_2N_2O_3S$ 641.0109, found: 6411.0113.

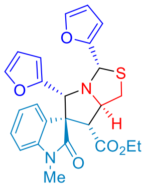

Compound ***5b:*** off-white solid (49% yield). ^{1}H NMR (400 MHz, $CDCl_3$) δ 7.49 – 7.45 (m, 1H), 7.36 (dt, J = 1.7, 0.7 Hz, 1H), 7.20 – 7.15 (m, 1H), 7.01 – 6.93 (m, 2H), 6.67 (d, J = 7.8 Hz, 1H), 6.35 – 6.29 (m, 2H), 6.00 (dt, J = 3.3, 0.9 Hz, 1H), 5.96 (dd, J = 3.3, 1.8 Hz, 1H), 5.35 (s, 1H), 4.78 – 4.71 (m, 2H), 3.80 (dd, J = 10.8, 7.1 Hz, 1H), 3.65 (dd, J = 10.8, 7.2 Hz, 1H), 3.54 (d, J = 8.6 Hz, 1H), 3.40 (dd, J = 12.1, 7.3 Hz, 1H), 3.20 (s, 3H), 3.02 (dd, J = 12.1, 1.8 Hz, 1H), 0.70 (t, J = 7.1 Hz, 3H). ^{13}C NMR (101 MHz, $CDCl_3$) δ 174.7, 169.0, 153.4, 151.5, 142.3, 142.0, 128.7, 126.5, 125.3, 122.2, 110.1, 109.8, 107.7, 107.5, 107.0, 69.6, 69.2, 67.5, 60.7, 59.8, 56.2, 38.9, 26.6, 13.5. HRMS (ESI-TOF*, m/z*): $[M+H]^+$ calcd. for $C_{25}H_{24}N_2O_5S$ 465.1484, found: 465.1479.

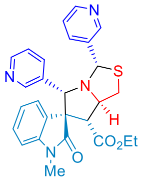

Compound ***5c:*** off-white solid (55% yield). ^{1}H NMR (400 MHz, $CDCl_3$) δ 8.77 – 8.68 (m, 2H), 8.46 (dd, J = 4.8, 1.5 Hz, 1H), 8.41 (dd, J = 4.8, 1.6 Hz, 1H), 7.87 (d, J = 8.0 Hz, 1H), 7.73 (d, J = 0.7 Hz, 1H), 7.67 – 7.61 (m, 1H), 7.30 (t, J = 7.8 Hz, 1H), 7.18 (ddd, J = 11.6, 7.9, 4.8 Hz, 2H), 7.09 (t, J = 7.6 Hz, 1H), 6.87 – 6.80 (m, 1H), 5.49 (s, 1H), 5.09 (d, J = 8.6 Hz, 1H), 4.33 (t, J = 7.6 Hz, 1H), 3.92 (d, J = 8.6 Hz, 1H), 3.51 (ddd, J = 7.1, 2.8, 0.7 Hz, 2H), 3.47 – 3.41 (m, 1H), 3.25 (s, 3H), 2.89 (dd, J = 11.6, 7.5 Hz, 1H), 0.61 (t, J = 7.2 Hz, 3H).^{13}C NMR (101 MHz, $CDCl_3$) δ174.8, 169.1, 143.7, 140.8, 135.3, 131.3, 133.0, 128.9, 128.9, 128.2, 126.0, 125.2, 122.3, 121.8, 121.2, 107.9, 75.3, 74.7, 67.5, 61.3, 60.7, 56.2, 38.8, 26.4, 13.5. HRMS (ESI-TOF*, m/z*): $[M+H]^+$ calcd. for $C_{27}H_{26}N_4O_3S$ 487.1804, found: 487.1807.

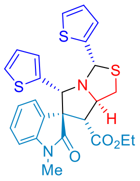

Compound ***5d:*** white solid (61% yield). ^{1}H NMR (400 MHz, $CDCl_3$) δ 7.94 – 7.88 (m, 1H), 7.43 (dt, J = 1.8, 0.9 Hz, 1H), 7.31 – 7.25 (m, 2H), 7.09 (tt, J = 7.6, 0.9 Hz, 1H), 6.82 (dq, J = 7.7, 0.8 Hz, 1H), 6.54 (dq, J = 3.3, 0.8 Hz, 1H), 6.42 – 6.37 (m, 1H), 6.20 – 6.13 (m, 2H), 5.52 (s, 1H), 4.63 – 4.59 (m, 1H), 4.17 (d, J = 10.8 Hz, 1H), 3.77 (dd, J = 9.8, 6.7 Hz, 1H), 3.68 (d, J = 0.8 Hz, 1H), 3.61 – 3.50 (m, 2H), 3.47 (d, J = 0.8 Hz, 1H),

3.25 (d, J = 0.9 Hz, 3H), 2.95 (ddd, J = 10.9, 6.7, 0.8 Hz, 1H), 0.66 – 0.60 (m, 3H).^{13}C NMR (101 MHz, $CDCl_3$) δ 175.4, 167.5, 155.0, 152.3, 142.9, 142.5, 142.3, 131.3, 128.5, 125.6, 123.0, 110.4, 110.0, 108.7, 107.6, 106.1, 74.6, 67.5, 60.3, 59.2, 56.9, 52.1, 32.8, 26.7, 13.4. HRMS (ESI-TOF, *m/z*): $[M+H]^+$ calcd. for $C_{25}H_{24}N_2O_3S_3$ 497.1027, found: 497.1031.

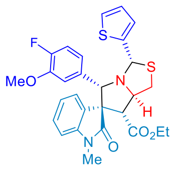

Compound ***7a****:* white solid (66% yield). ^{1}H NMR (400 MHz, $CDCl_3$) δ 7.67 – 7.62 (m, 1H), 7.24 (s, 1H), 7.21 (dd, J = 7.8, 1.2 Hz, 1H), 7.19 – 7.13 (m, 1H), 7.08 – 7.00 (m, 2H), 6.91 (dd, J = 5.1, 1.2 Hz, 1H), 6.82 – 6.78 (m, 1H), 6.69 – 6.62 (m, 2H), 5.34 (s, 1H), 5.02 (s, 1H), 4.71 – 4.66 (m, 1H), 3.91 (s, 3H), 3.85 – 3.79 (m, 1H), 3.65 (dd, J = 10.8, 7.1 Hz, 1H), 3.57 (d, J = 8.6 Hz, 1H), 3.12 (d, J = 13.2 Hz, 4H), 2.97 (dd, J = 12.1, 1.7 Hz, 1H), 0.69 (t, J = 7.1 Hz, 3H).^{13}C NMR (101 MHz, $CDCl_3$) δ 174.7, 169.1, 144.1, 140.1, 137.4, 137.4, 129.0, 126.4, 125.9, 125.9, 125.6, 125.6, 122.2, 119.1, 119.1, 115.3, 115.1, 112.5, 112.4, 107.8, 75.3, 71.5, 66.8, 61.2, 60.7, 56.1, 55.9, 39.0, 26.5, 13.5. HRMS (ESI-TOF, *m/z*): $[M+H]^+$ calcd. for $C_{28}H_{27}FN_2O_4S_2$ 539.1475, found: 539.1477.

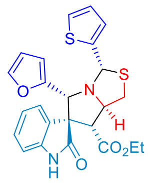

Compound ***7b****:* white solid (51% yield). ^{1}H NMR (400 MHz, $CDCl_3$) δ 7.62 – 7.57 (m, 1H), 7.19 (dd, J = 7.8, 1.3 Hz, 1H), 7.14 – 7.10 (m, 1H), 7.06 – 7.00 (m, 2H), 6.98 – 6.94 (m, 1H), 6.71 – 6.65 (m, 1H), 6.11 (d, J = 3.3 Hz, 1H), 5.97 (dd, J = 3.3, 1.8 Hz, 1H), 5.51 (s, 1H), 4.79 (s, 1H), 4.72 – 4.67 (m, 1H), 3.80 (dd, J = 10.8, 7.1 Hz, 1H), 3.65 (dd, J = 10.8, 7.1 Hz, 1H), 3.53 (d, J = 8.6 Hz, 1H), 3.31 (s, 1H), 3.19 (s, 3H), 3.01 (dd, J = 12.1, 1.7 Hz, 1H), 0.70 (t, J = 7.1 Hz, 3H).^{13}C NMR (101 MHz, $CDCl_3$) δ 174.6, 169.0, 151.3, 147.2, 143.9, 142.0, 128.7, 127.2, 126.6, 125.4, 125.3, 125.0, 122.3, 109.9, 107.7, 107.5, 71.9, 68.8, 66.8, 60.7, 59.8, 56.1, 39.4, 26.6, 13.5. HRMS (ESI-TOF, *m/z*): $[M+H]^+$ calcd. for $C_{25}H_{24}N_2O_4S_2$ 481.1256, found: 481.1261.

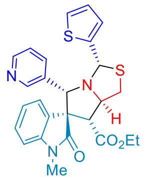

Compound ***7c****:* white solid (43% yield). ^{1}H NMR (400 MHz, $CDCl_3$) δ 8.44 (dd, J = 2.3, 0.7 Hz, 1H), 8.26 (dd, J = 4.8, 1.7 Hz, 1H), 7.69 – 7.65 (m, 1H), 7.62 – 7.56 (m, 1H), 7.24 – 7.22 (m, 1H), 7.18 – 7.12 (m, 1H), 7.10 (ddt, J = 3.4, 1.4, 0.7 Hz, 1H), 7.08 – 7.02 (m, 1H), 6.98 – 6.90 (m, 2H), 6.55 (dd, J = 7.8, 1.0 Hz, 1H), 5.32 (d, J = 1.4 Hz, 1H), 4.85 – 4.80 (m, 1H), 4.75 (s, 1H), 3.82 (dd, J = 10.8, 7.1 Hz, 1H), 3.69 – 3.63 (m, 1H), 3.61 (d, J = 8.6 Hz, 1H), 3.34 (dd, J = 12.1, 7.4 Hz, 1H), 3.10 (s, 3H), 3.06 (dd, J = 12.2, 1.7 Hz, 1H), 0.67 (t, J = 7.1 Hz, 3H).^{13}C NMR (101 MHz, $CDCl_3$) δ 174.4, 169.0, 149.5, 149.1, 146.6, 143.7, 135.1, 131.7, 129.0, 127.3, 125.7, 125.3, 125.2, 125.1, 122.7, 122.5, 107.8, 72.5, 71.7, 67.1, 61.3, 60.7, 56.2, 39.7, 26.4, 13.5. HRMS (ESI-TOF, *m/z*): $[M+H]^+$ calcd. for $C_{26}H_{25}N_3O_3S_2$ 492.1416, found: 492.1413.

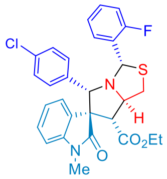

*Compound **7d**:* white solid (72% yield). ^{1}H NMR (400 MHz, $CDCl_3$) δ 7.61 – 7.56 (m, 1H), 7.53 – 7.43 (m, 2H), 7.20 (dd, J = 7.4, 1.5 Hz, 1H), 7.13 (td, J = 7.8, 1.3 Hz, 1H), 7.03 – 6.95 (m, 4H), 6.92 – 6.88 (m, 2H), 6.58 – 6.55 (m, 1H), 5.33 (dd, J = 11.3, 1.8 Hz, 1H), 4.98 – 4.94 (m, 1H), 4.77 (s, 1H), 3.83 (td, J = 7.1, 3.6 Hz, 1H), 3.69 – 3.62 (m, 2H), 3.28 – 3.23 (m, 1H), 3.12 (s, 3H), 3.06 (dd, J = 12.3, 1.7 Hz, 1H), 0.70 (t, J = 7.1 Hz, 3H).^{13}C NMR (101 MHz, $CDCl_3$) δ 174.8, 169.2, 144.0, 143.7, 134.9, 133.4, 128.9, 128.8, 128.6, 128.5, 128.0, 126.7, 126.7, 125.9, 125.2, 125.1, 123.7, 123.6, 122.2, 121.8, 115.7, 115.5, 107.8, 107.5, 74.8, 69.9, 69.9, 68.7, 61.4, 60.7, 60.7, 56.2, 56.0, 38.2, 38.1, 26.5, 26.4, 13.5, 13.5. HRMS (ESI-TOF*, m/z*): $[M+H]^+$ calcd. for $C_{29}H_{26}ClFN_2O_3S$ 537.1415, found: 537.1418.

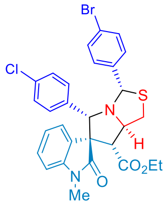

*Compound **7e**:* white solid (66% yield). ^{1}H NMR (400 MHz, $CDCl_3$) δ 7.68 – 7.65 (m, 1H), 7.62 – 7.58 (m, 2H), 7.44 (d, J = 2.0 Hz, 1H), 7.42 (d, J = 1.6 Hz, 1H), 7.35 – 7.33 (m, 3H), 7.32 (t, J = 1.2 Hz, 1H), 7.27 – 7.25 (m, 1H), 7.10 (dtd, J = 15.3, 7.6, 1.1 Hz, 2H), 6.85 (dt, J = 7.7, 0.9 Hz, 1H), 5.34 (s, 1H), 4.70 (d, J = 11.1 Hz, 1H), 4.45 (dd, J = 8.0, 2.4 Hz, 1H), 3.72 – 3.66 (m, 2H), 3.60 – 3.49 (m, 2H), 3.25 (s, 3H), 2.61 (dd, J = 12.3, 7.9 Hz, 1H), 2.45 (dd, J = 12.2, 2.5 Hz, 1H), 0.61 (t, J = 7.1 Hz, 3H).^{13}C NMR (101 MHz, $CDCl_3$) δ 13C NMR (101 MHz, cdcl3) δ 177.5, 168.0, 144.0, 142.2, 140.9, 139.2, 134.5, 131.7, 131.4, 131.2, 131.1, 130.4, 129.4, 129.3, 129.0, 128.5, 128.4, 126.5, 125.5, 123.7, 123.0, 122.4, 122.2, 121.1, 108.1, 108.0, 74.5, 73.1, 69.4, 60.4, 60.0, 59.2, 52.6, 36.0, 26.8, 13.4. HRMS (ESI-TOF*, m/z*): $[M+H]^+$ calcd. for $C_{29}H_{26}BrClN_2O_3S$ 597.0614, found: 597.0611.

4. NMR **spectra of products**

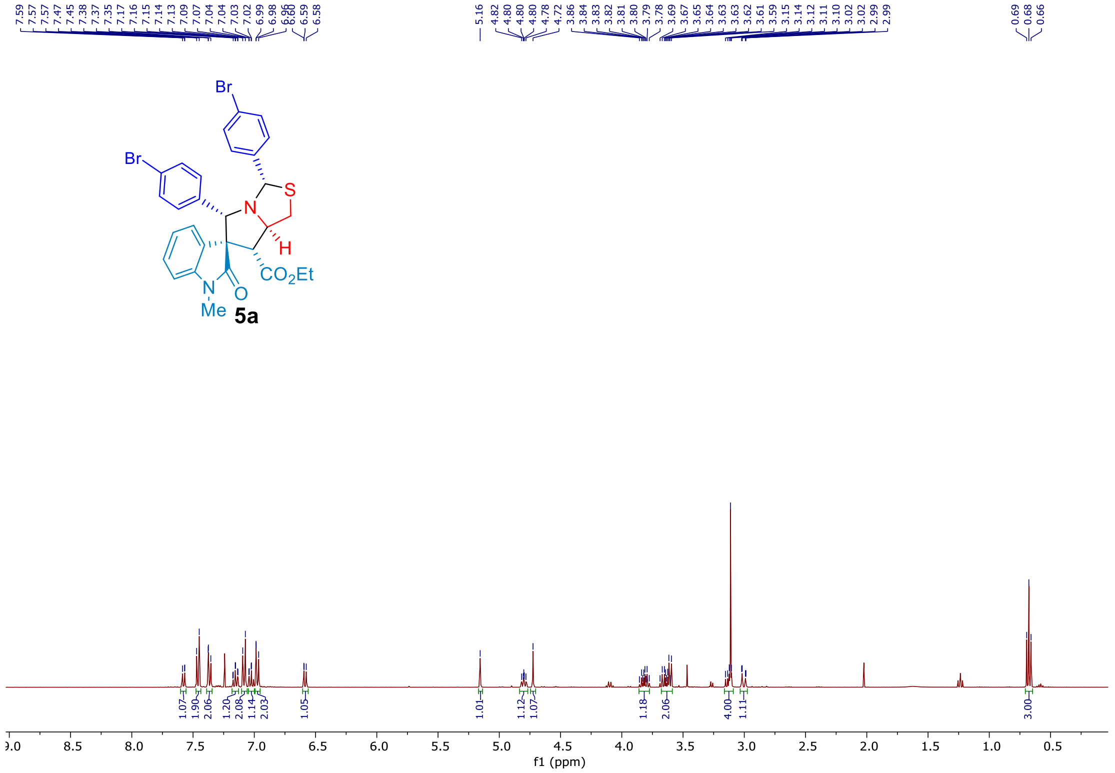

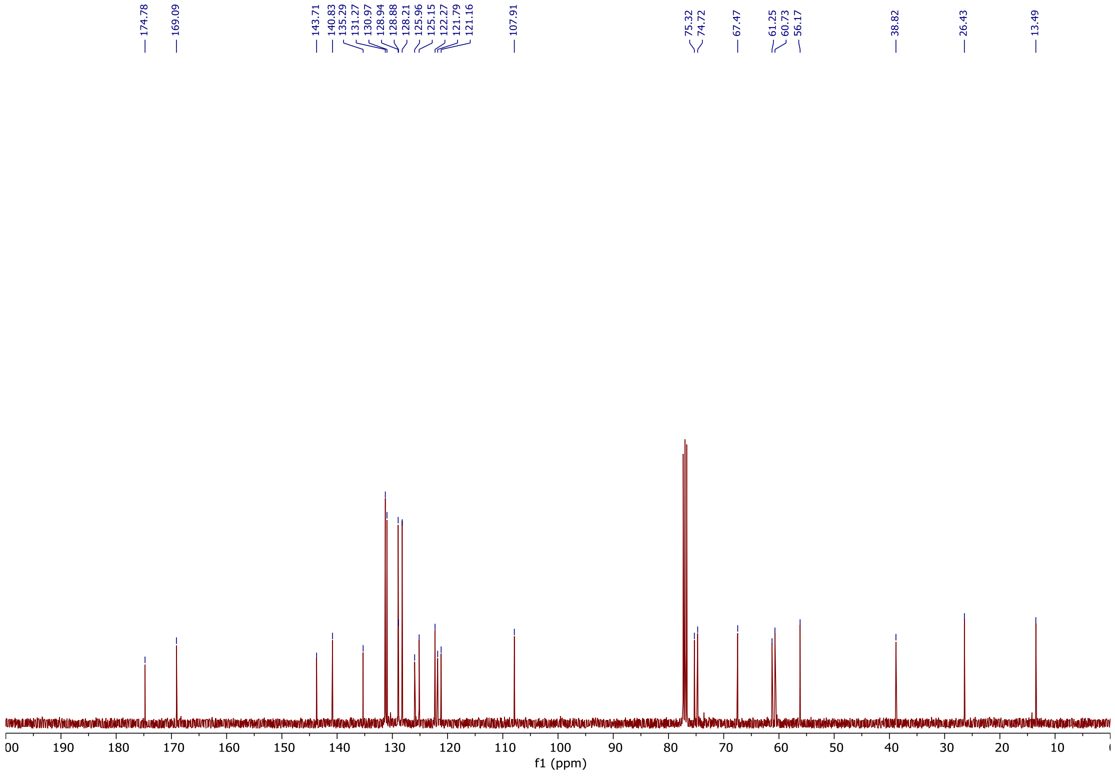

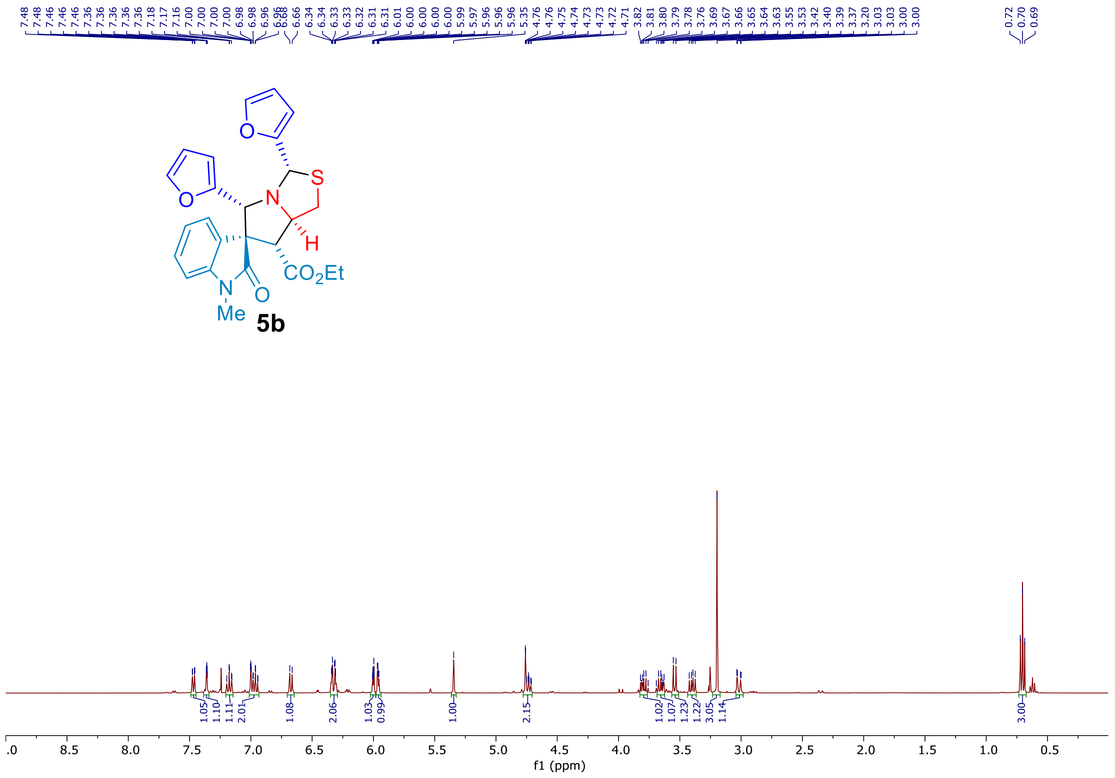
5b
f1 (ppm)

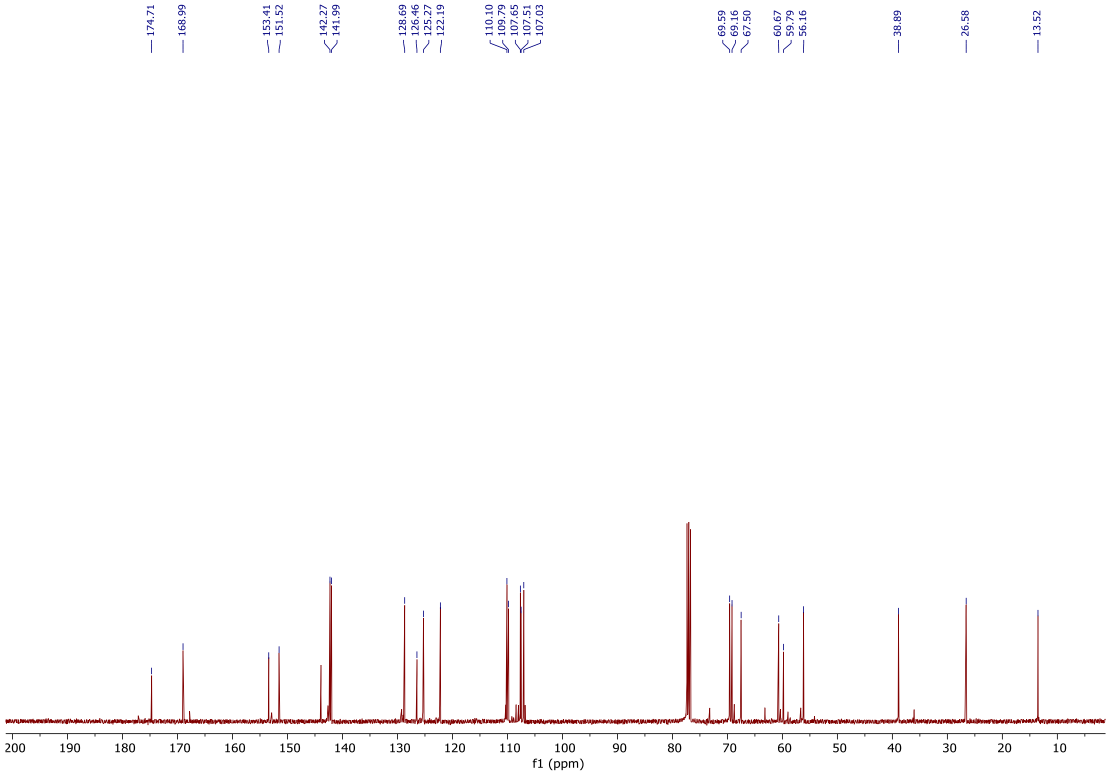
174.71
168.99
153.41
151.52
142.27
141.99
128.69
126.46
125.27
122.19
110.10
109.79
107.65
107.51
107.03
69.59
69.16
67.50
60.67
59.79
56.16
38.89
26.58
13.52
f1 (ppm)

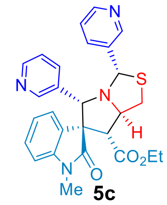
N
N
S
N
H
N
O
CO2Et
Me

5c

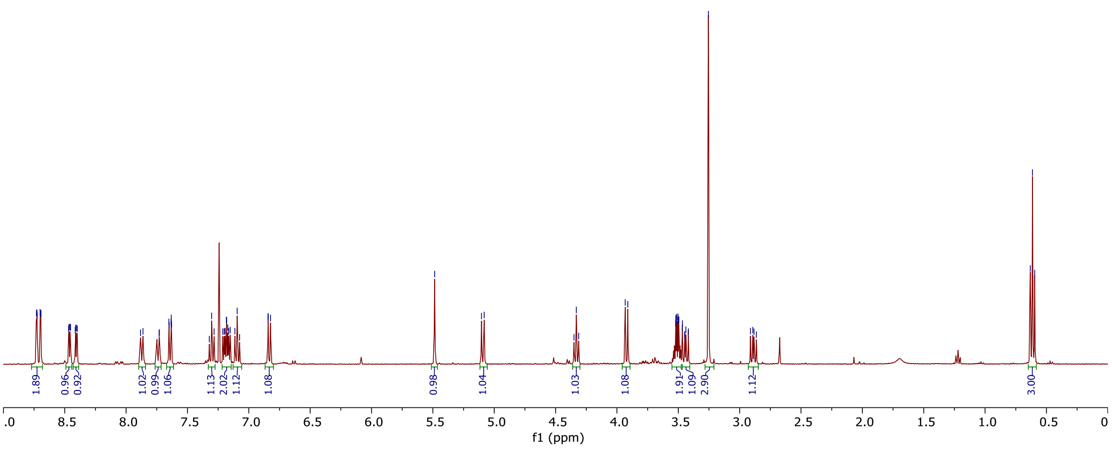
8.73 8.73 8.72 8.70 8.70 8.69 8.47 8.46 8.46 8.45 8.41 8.41 8.40 8.40 7.88 7.86 7.73 7.73 7.65 7.63 7.63 7.63 7.32 7.30 7.28 7.21 7.20 7.19 7.18 7.18 7.17 7.16 7.15 7.11 7.09 7.08 6.84 6.84 6.82 5.49 5.10 5.08
4.35 4.33 4.31
3.93 3.91 3.52 3.52 3.51 3.51 3.50 3.50 3.50 3.50 3.47 3.45 3.45 3.44 3.42 3.42 3.25 2.91 2.89 2.88 2.86
0.63 0.61 0.60
1.89 0.96 0.92 1.02 0.99 1.06 1.13 2.02 1.12 1.08 0.98 1.04 1.03 1.08 1.91 1.09 2.90 1.12 3.00
f1 (ppm)

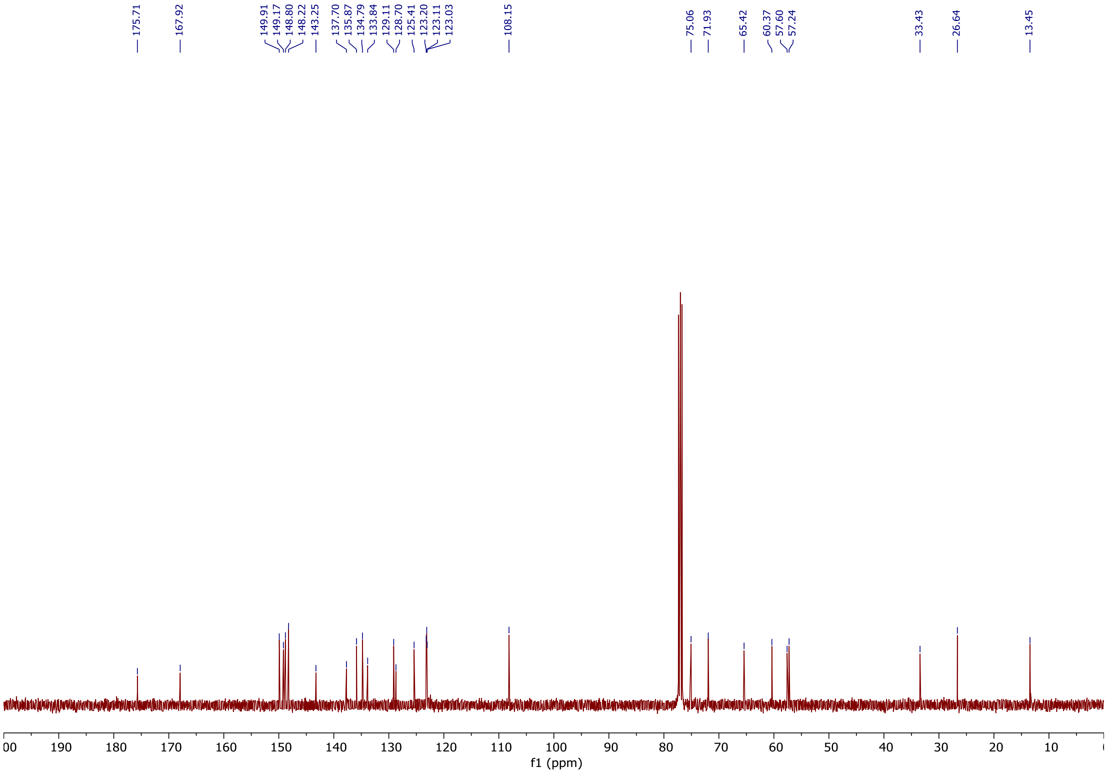
175.71
167.92
149.91 149.17 148.80 148.22 143.25 137.70 135.87 134.79 133.84 129.11 128.70 125.41 123.20 123.11 123.03
108.15
75.06 71.93 65.42 60.37 57.60 57.24
33.43 26.64 13.45
f1 (ppm)

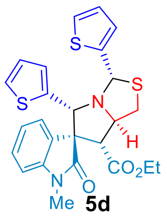

5d

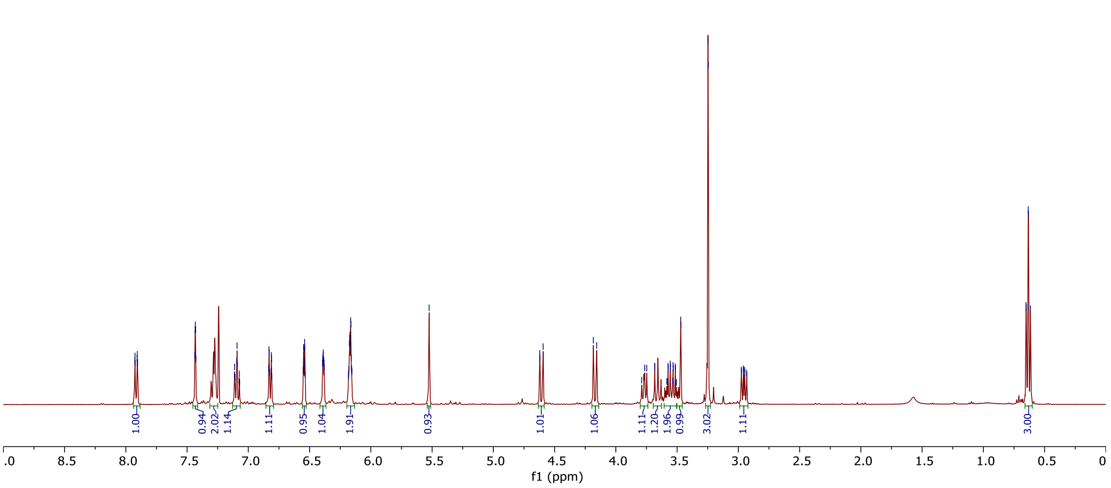
7.93 7.92 7.91 7.91 7.90 7.44 7.43 7.43 7.43 7.43 7.11 7.11 7.09 7.09 7.09 7.07 6.83 6.83 6.83 6.81 6.81 6.81 6.55 6.55 6.55 6.54 6.54 6.54 6.54 6.39 6.39 6.39 6.38 6.38 6.38 6.18 6.18 6.18 6.17 6.17 6.17 6.17 6.16 6.16 6.16 6.15 5.52 4.62 4.62 4.59 4.18 4.16 3.76 3.75 3.68 3.57 3.57 3.56 3.55 3.53 3.53 3.52 3.51 3.47 3.25 3.25 2.98 2.97 2.96 2.96 2.95 2.95 2.93 2.93 0.65 0.65 0.63 0.63 0.62
1.00 0.94 2.02 1.14 1.11 0.95 1.04 1.91 0.93 1.01 1.06 1.11 1.20 1.96 0.99 3.02 1.11 3.00
f1 (ppm)

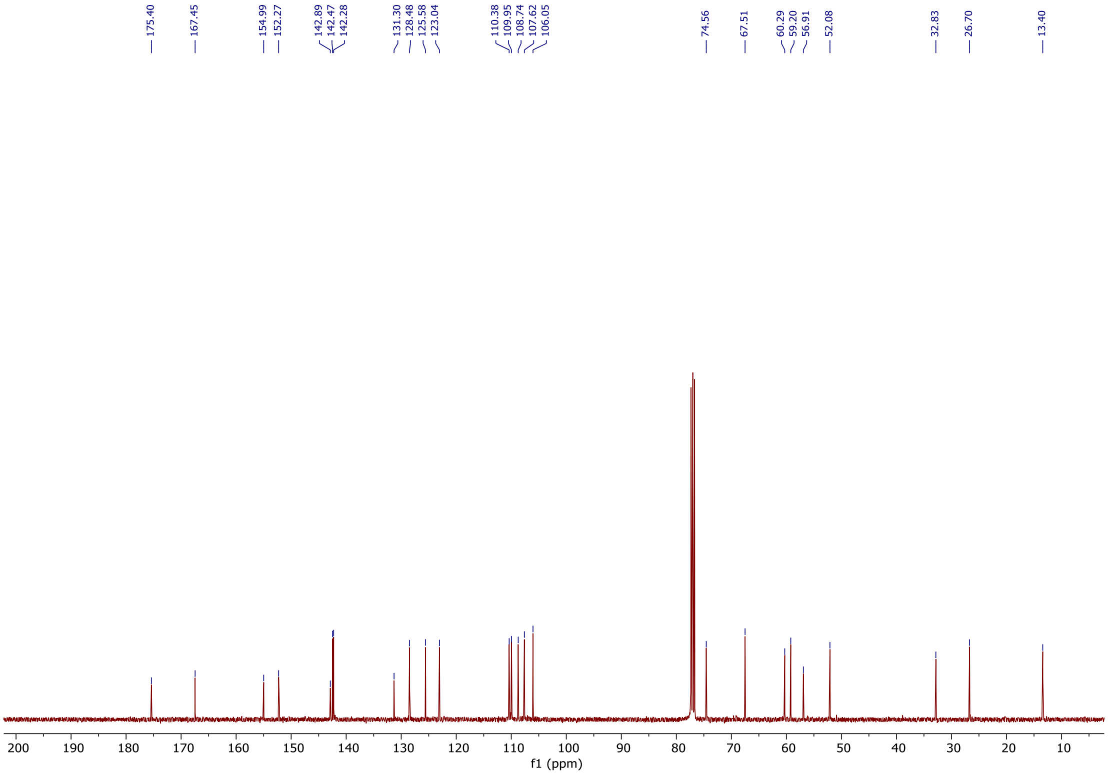
175.40 167.45 154.99 152.27 142.89 142.47 142.28 131.30 128.48 125.58 123.04 110.38 109.95 108.74 107.62 106.05 74.56 67.51 60.29 59.20 56.91 52.08 32.83 26.70 13.40
f1 (ppm)

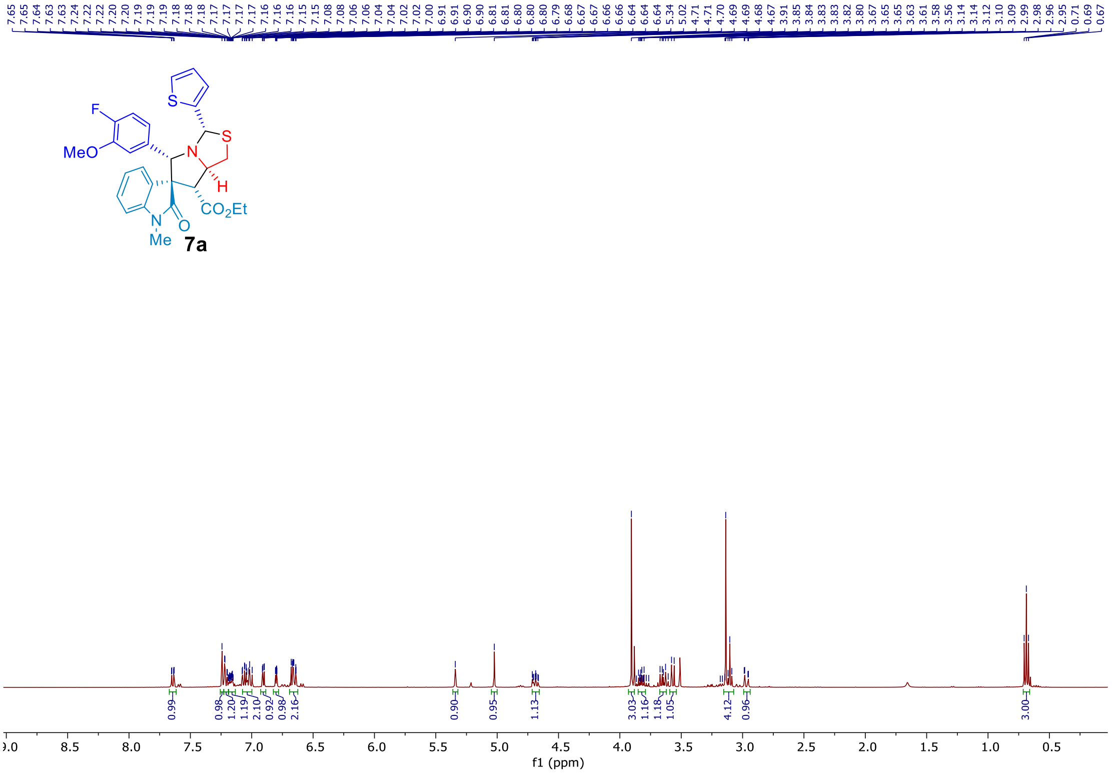
F
MeO
S
S
N
H
CO2Et
N
Me
O

7a

7.65 7.65 7.64 7.63 7.63 7.24 7.22 7.22 7.20 7.20 7.19 7.19 7.19 7.18 7.18 7.18 7.17 7.17 7.17 7.17 7.16 7.16 7.16 7.15 7.15 7.08 7.08 7.06 7.06 7.04 7.04 7.02 7.02 7.00 6.91 6.91 6.90 6.90 6.81 6.81 6.80 6.80 6.79 6.68 6.67 6.67 6.66 6.66 6.64 6.64 6.64 5.34 5.02 4.71 4.71 4.70 4.69 4.69 4.68 4.67 3.91 3.85 3.84 3.83 3.83 3.82 3.80 3.67 3.65 3.65 3.63 3.61 3.58 3.56 3.14 3.14 3.12 3.10 3.09 2.99 2.98 2.96 2.95 0.71 0.69 0.67
0.99 0.98 1.20 1.19 2.10 0.92 0.98 2.16 0.90 0.95 1.13 3.03 1.16 1.18 1.05 4.12 0.96 3.00
8.5 8.0 7.5 7.0 6.5 6.0 5.5 5.0 4.5 4.0 3.5 3.0 2.5 2.0 1.5 1.0 0.5
f1 (ppm)

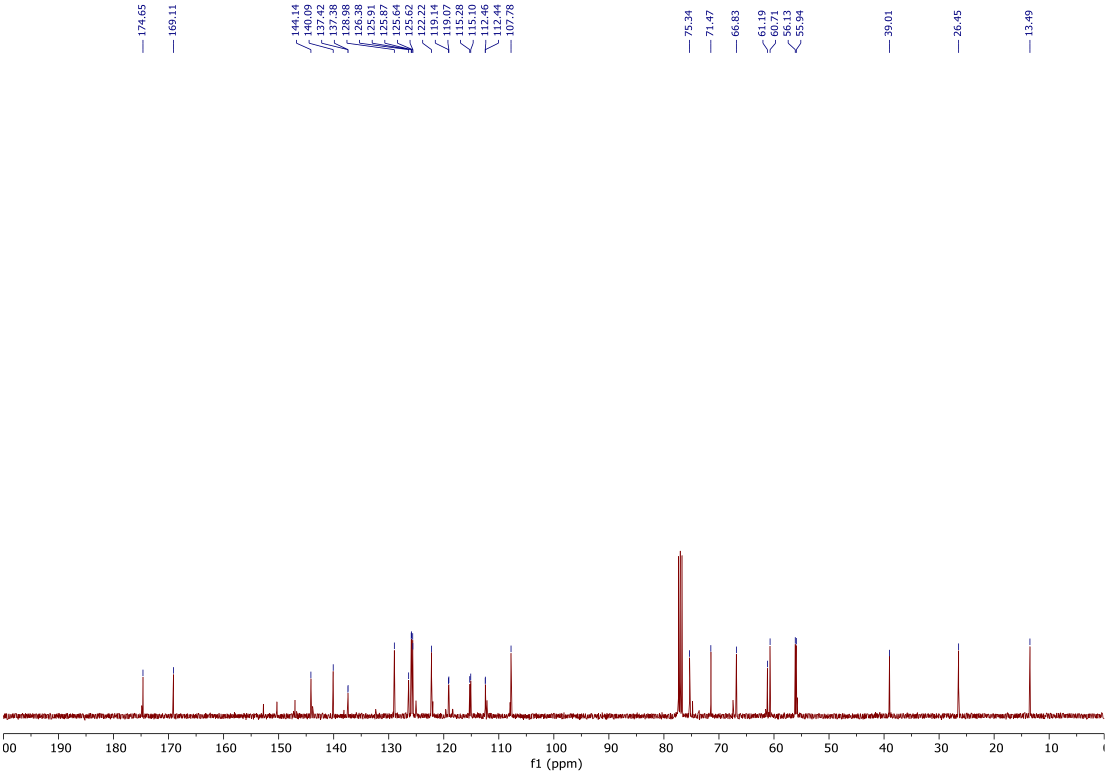
174.65 169.11 144.14 140.09 137.42 137.38 128.98 126.38 125.91 125.87 125.64 125.62 122.22 119.14 119.07 115.28 115.10 112.46 112.44 107.78 75.34 71.47 66.83 61.19 60.71 56.13 55.94 39.01 26.45 13.49
190 180 170 160 150 140 130 120 110 100 90 80 70 60 50 40 30 20 10
f1 (ppm)

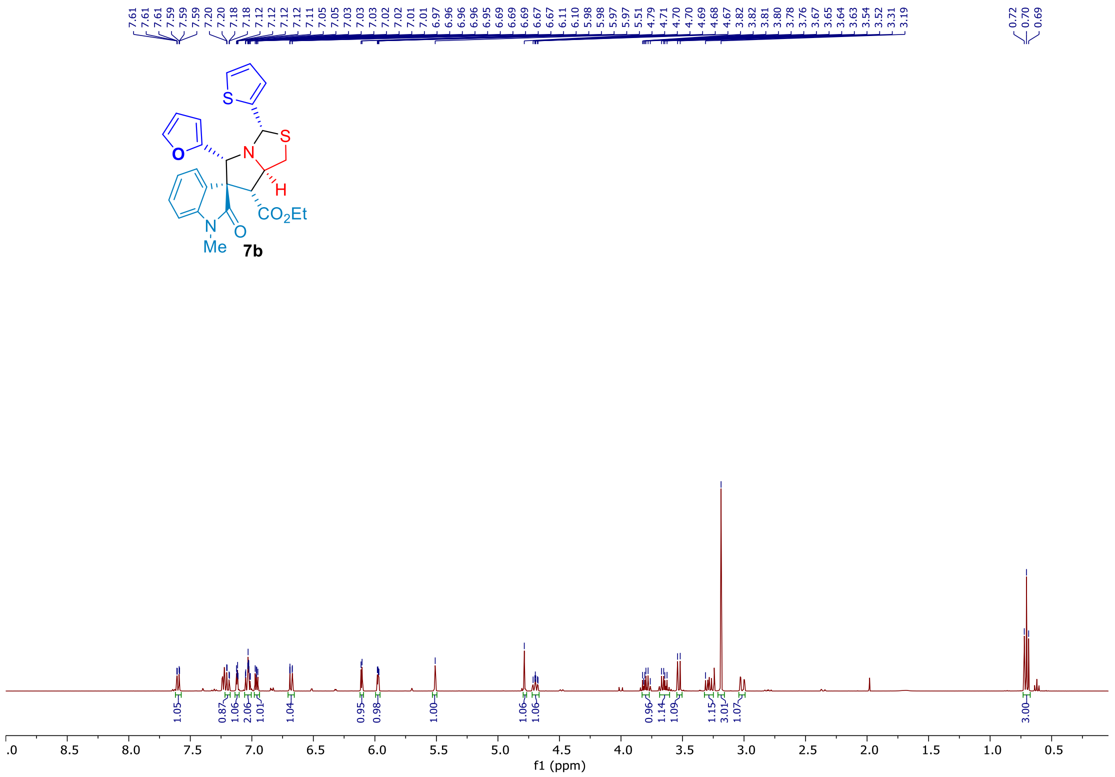
7b
N
S
O
H
CO2Et
Me
f1 (ppm)

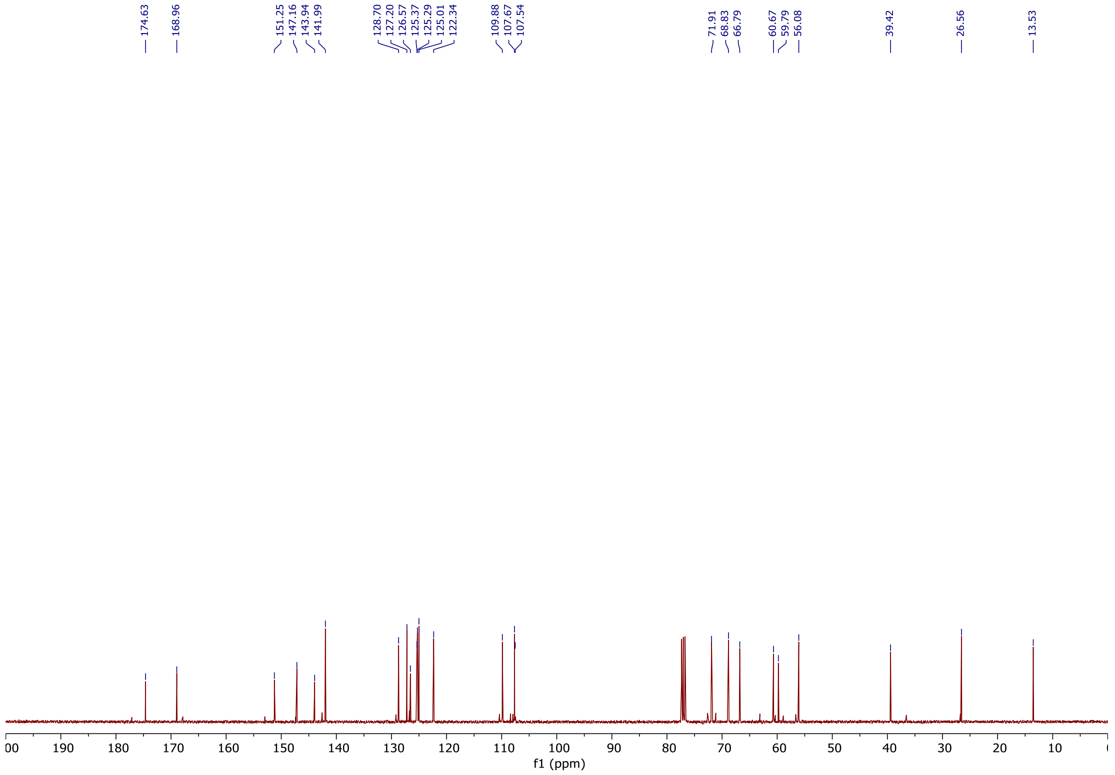
174.63
168.96
151.25
147.16
143.94
141.99
128.70
127.20
126.57
125.37
125.29
125.01
122.34
109.88
107.67
107.54
71.91
68.83
66.79
60.67
59.79
56.08
39.42
26.56
13.53
f1 (ppm)

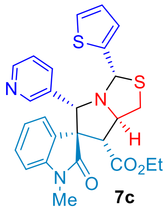
S
N
S
N
H
CO2Et
N
Me
O

7c

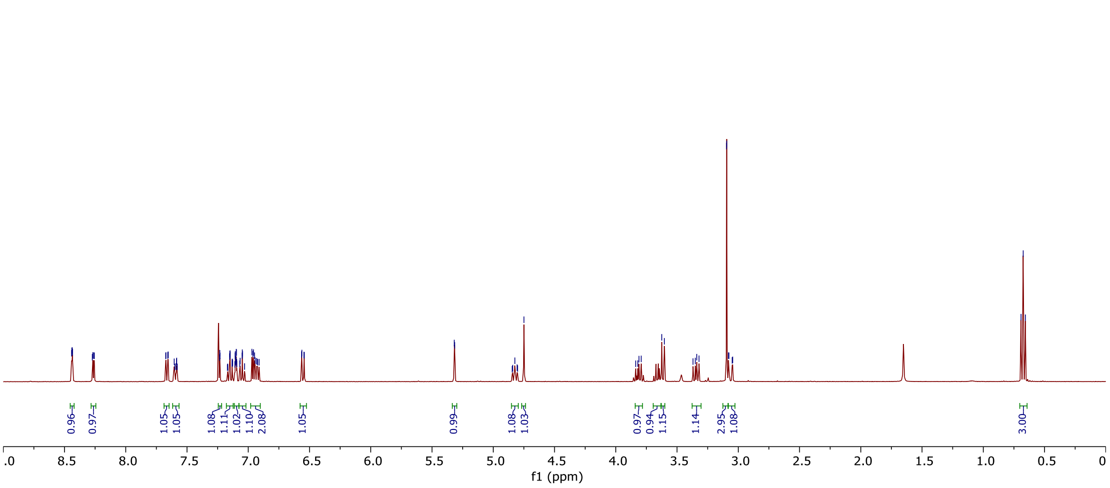
8.44 8.44 8.44 8.43 8.27 8.27 8.26 8.26 7.67 7.67 7.66 7.65 7.60 7.60 7.59 7.59 7.58 7.58 7.23 7.23 7.23 7.17 7.17 7.17 7.15 7.15 7.15 7.13 7.13 7.13 7.11 7.11 7.11 7.10 7.10 7.10 7.10 7.09 7.09 7.07 7.07 7.05 7.05 7.05 7.03 6.97 6.96 6.96 6.95 6.94 6.93 6.92 6.91 6.56 6.56 6.55 6.54 5.32 5.31 4.84 4.82 4.81 4.80 4.75 3.84 3.82 3.81 3.79 3.62 3.60 3.37 3.35 3.34 3.32 3.10 3.09 3.08 3.08 3.05 0.69 0.67 0.66
0.96 0.97 1.05 1.05 1.08 1.11 1.02 1.10 2.08 1.05 0.99 1.08 1.03 0.97 0.94 1.15 1.14 2.95 1.08 3.00
.0 8.5 8.0 7.5 7.0 6.5 6.0 5.5 5.0 4.5 4.0 3.5 3.0 2.5 2.0 1.5 1.0 0.5 0
f1 (ppm)

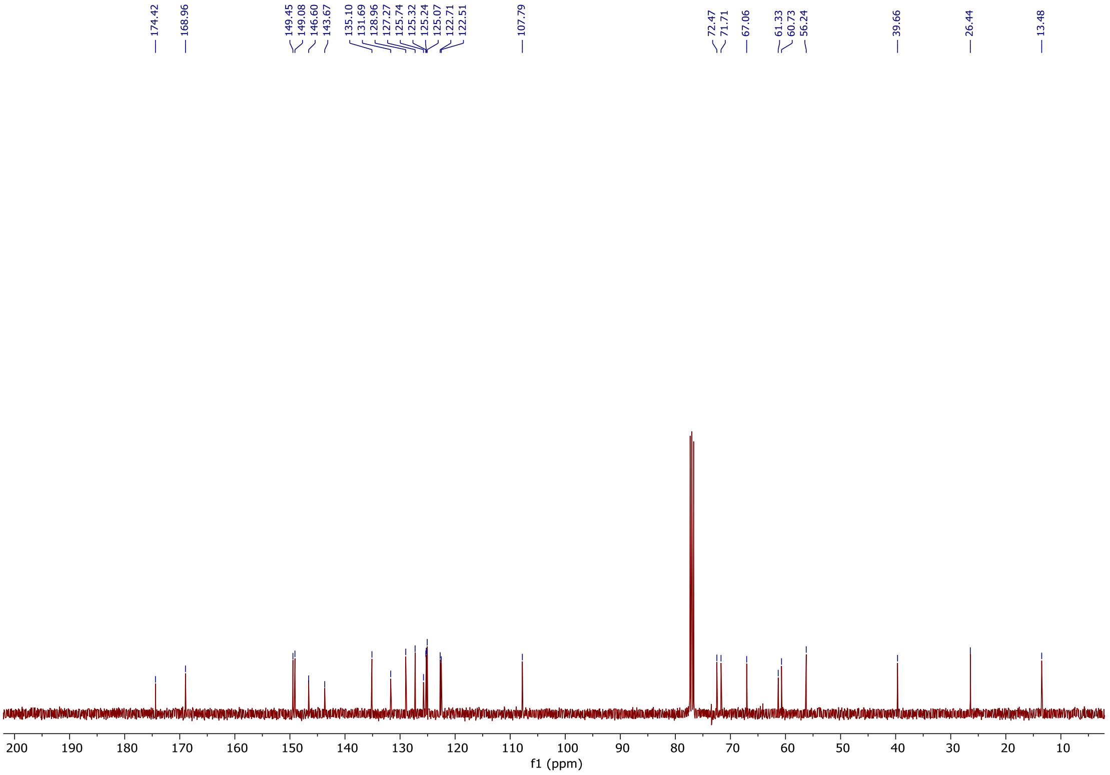
174.42 168.96 149.45 149.08 146.60 143.67 135.10 131.69 128.96 127.27 125.74 125.32 125.24 125.07 122.71 122.51 107.79 72.47 71.71 67.06 61.33 60.73 56.24 39.66 26.44 13.48
200 190 180 170 160 150 140 130 120 110 100 90 80 70 60 50 40 30 20 10
f1 (ppm)

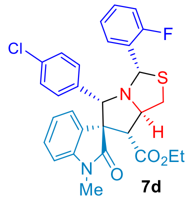
Cl
F
S
N
H
CO2Et
N
Me
O

7d

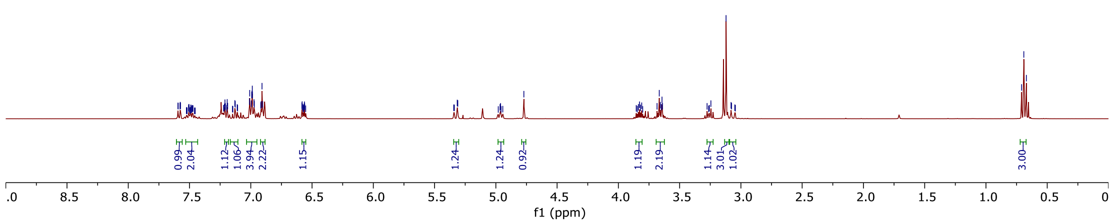
7.59
7.59
7.58
7.58
7.57
7.51
7.51
7.50
7.50
7.49
7.49
7.49
7.48
7.47
7.47
7.22
7.22
7.21
7.21
7.20
7.19
7.19
7.15
7.15
7.13
7.13
7.11
7.11
7.01
7.01
7.00
6.99
6.99
6.99
6.99
6.97
6.97
6.92
6.92
6.91
6.90
6.58
6.58
6.58
6.57
6.57
6.56
6.56
6.56
6.55
5.34
5.34
5.32
5.31
4.98
4.97
4.96
4.96
4.94
4.77
3.85
3.85
3.84
3.83
3.82
3.81
3.80
3.68
3.67
3.66
3.65
3.64
3.64
3.28
3.26
3.26
3.24
3.12
3.08
3.08
3.05
3.05
0.71
0.69
0.67
0.99
2.04
1.12
1.06
3.94
2.22
1.15
1.24
1.24
0.92
1.19
2.19
1.14
3.01
1.02
3.00
.0
8.5
8.0
7.5
7.0
6.5
6.0
5.5
5.0
4.5
4.0
3.5
3.0
2.5
2.0
1.5
1.0
0.5
0
f1 (ppm)

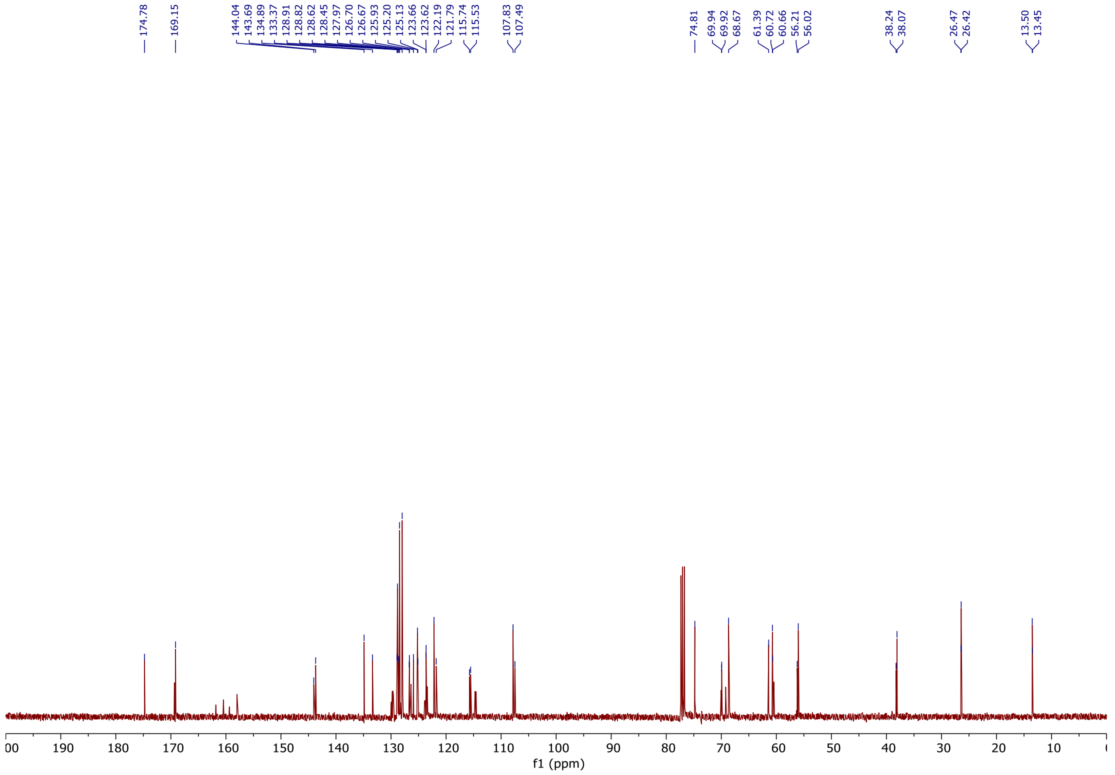
174.78
169.15
144.04
143.69
134.89
133.37
128.91
128.82
128.45
127.97
126.70
126.67
125.93
125.20
125.13
123.66
123.62
122.19
121.79
115.74
115.53
107.83
107.49
74.81
69.94
69.92
68.67
61.39
60.72
60.66
56.21
56.02
38.24
38.07
26.47
26.42
13.50
13.45
00
190
180
170
160
150
140
130
120
110
100
90
80
70
60
50
40
30
20
10
f1 (ppm)

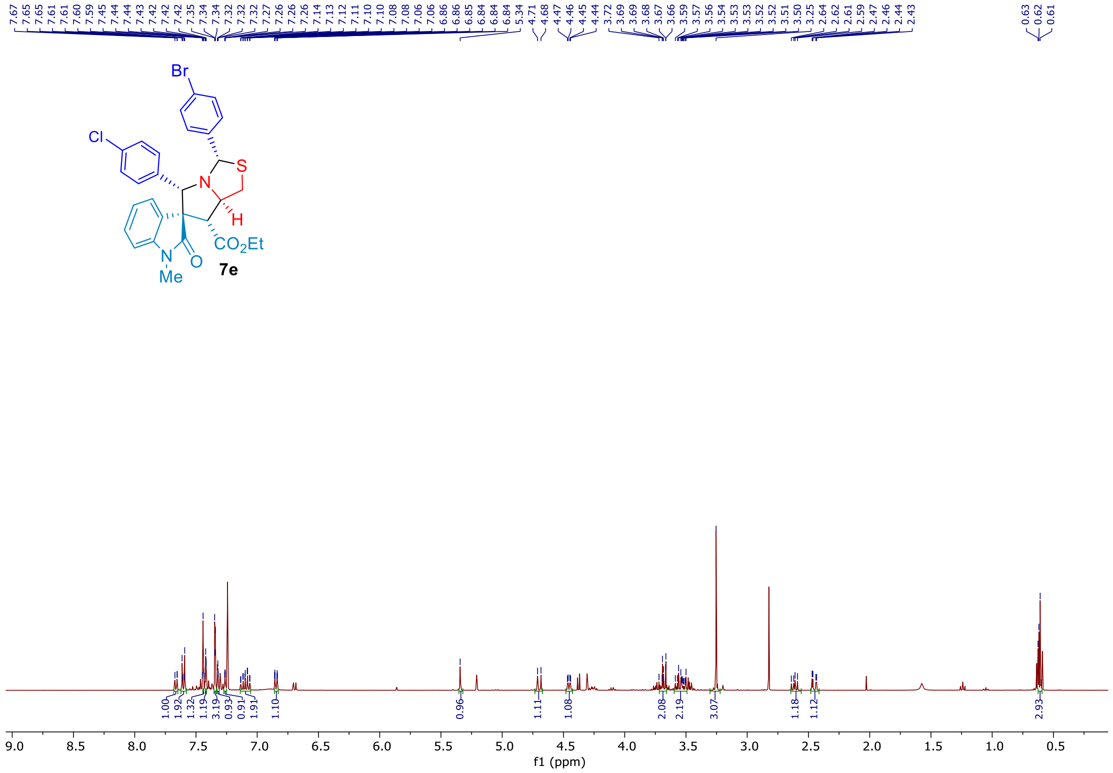

Br
Cl
N
S
H
CO_2Et
N
Me
O
7e
7.67 7.65 7.65 7.61 7.60 7.59 7.45 7.44 7.43 7.42 7.42 7.42 7.35 7.34 7.34 7.32 7.32 7.32 7.27 7.26 7.26 7.26 7.14 7.13 7.12 7.11 7.10 7.08 7.08 7.06 7.06 6.86 6.86 6.85 6.84 6.84 5.34 4.71 4.68 4.47 4.46 4.45 4.44 3.72 3.69 3.69 3.68 3.67 3.66 3.59 3.57 3.56 3.54 3.53 3.53 3.52 3.52 3.51 3.50 3.25 2.64 2.62 2.62 2.61 2.59 2.47 2.46 2.44 2.43
0.63 0.62 0.61
1.00 1.92 1.32 1.19 3.19 0.93 0.91 1.91 1.10 0.96 1.11 1.08 2.08 2.19 3.07 1.18 1.12 2.93
9.0 8.5 8.0 7.5 7.0 6.5 6.0 5.5 5.0 4.5 4.0 3.5 3.0 2.5 2.0 1.5 1.0 0.5
f1 (ppm)

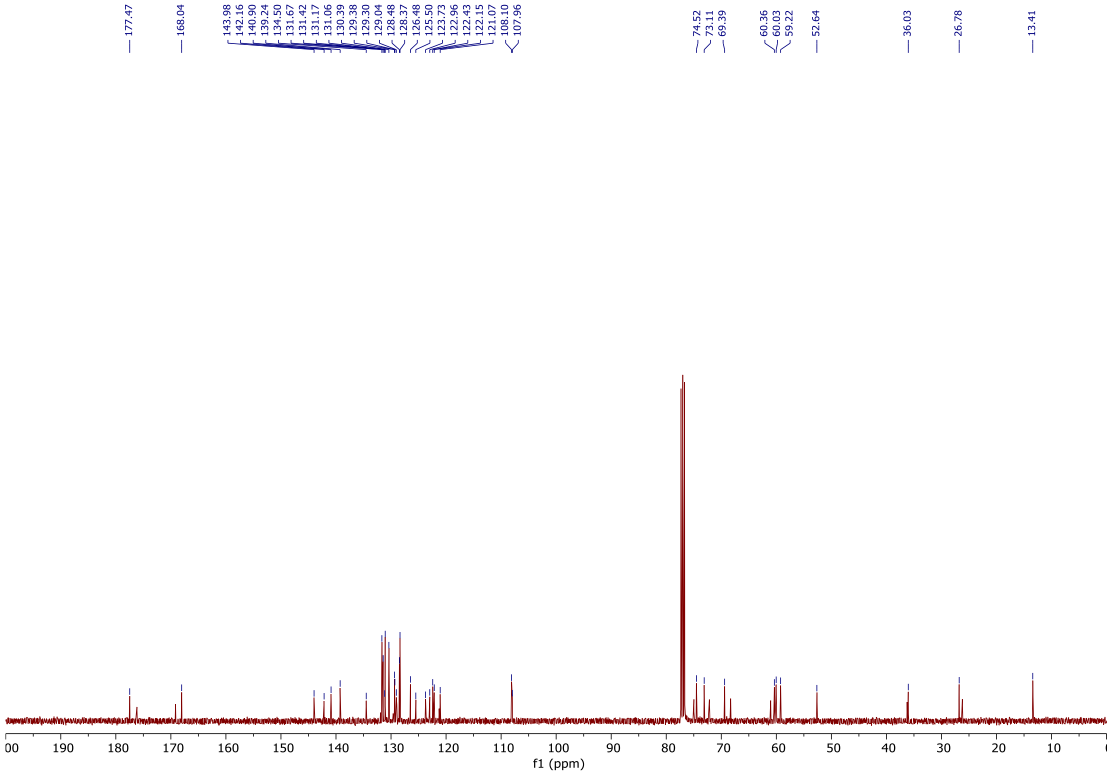

177.47
168.04
143.98 142.16 140.90 139.24 134.50 131.67 131.42 131.17 131.06 130.39 129.38 129.30 129.04 128.48 128.37 126.48 125.50 123.73 122.96 122.43 122.15 121.07 108.10 107.96
74.52 73.11 69.39
60.36 60.03 59.22
52.64
36.03
26.78
13.41
190 180 170 160 150 140 130 120 110 100 90 80 70 60 50 40 30 20 10
f1 (ppm)

5. Green chemistry metrics analysis

The following formulae were used for calculating Atom Economy (AE), Atom Efficiency (AEf), Carbon Efficiency (CE), Reaction Mass Efficiency (RME), Optimum Efficiency (OE), Mass Productivity (MP), Mass Intensity (MI) and Process Mass Intensity (PMI), E factor, Solvent and Water Intensity (SI and WI) [1-12].

$$AE = \frac{\text{Molecular weight of procuct}}{\text{Total molecular weight of reactants}} \times 100$$

$$AEf = \text{AE} \times \text{yield\%}$$

$$CE = \frac{\text{Amount of carbon in the product}}{\text{Total carbon present in reactants}} \times 100$$

$$RME = \frac{\text{Mass of isolated product}}{\text{Total mass of reactants}} \times 100$$

$$OE = \frac{RME}{AE} \times 100$$

$$MI = \frac{\textit{Total mass of input material in a process or process step}}{\textit{Mass of product}}$$

$$PMI = \frac{\text{Total mass of input material in the whole process}}{\text{Mass of product}}$$

$$MP = \frac{1}{PMI} \times 100$$

$$E\ Factor = PMI - 1$$

$$SI = \frac{\text{Total mass of solvents excl. water in the whole process}}{\text{Mass of product}}$$

$$WI = \frac{\text{Total mass of water used in the whole process}}{\text{Mass of product}}$$

5.1 Cascade process

*i. General procedure for the cascade synthesis of compounds **5a***

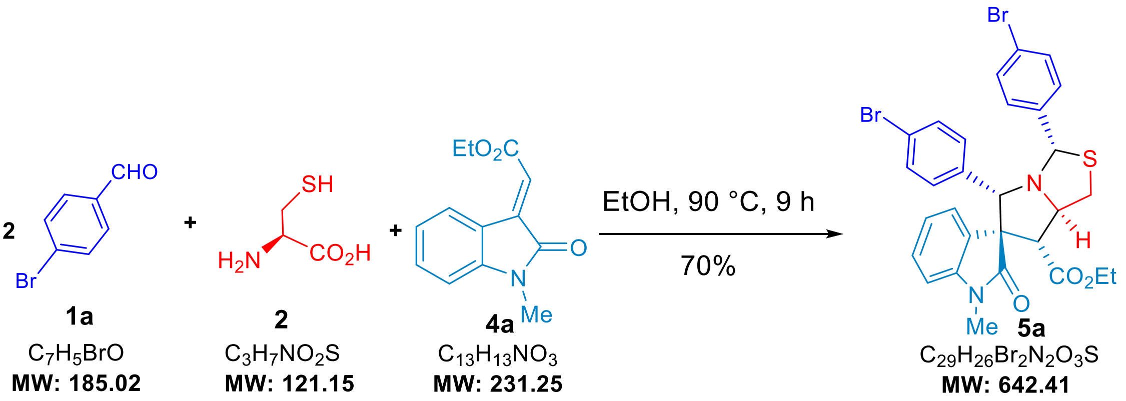

To a solution of 4-bromobenzaldehyde **1a** (2.2 mmol) and cysteine **2** (1.1 mmol), in 2.0 mL of EtOH was added olefinic oxindole **4a** (1.0 mmol). After being stirred at 90 °C for 9 h. Upon the completion of the reaction as monitored by LCMS, the concentrated reaction mixture was isolated on a semi-preparative HPLC with a C18 column (eluent, MeOH/H_2O = 70–90%). The product **5a** was afforded (70%).

Materials used for metrics calculations: 4-bromobenzaldehyde **1a** (407.0 mg, 2.2 mmol), cysteine **2** (133.3 mg, 1.1 mmol) and olefinic oxindole **4a** (231.3 mg, 1.0 mmol), EtOH (1578 mg, 2 mL), and product **5a** 449.7 mg (0.70 mmol).

$$AE(5a) = \frac{642.41}{185.02 \times 2 + 121.15 + 231.25} \times 100 = 88.9$$

$$AEf(5a) = 88.9 \times 70\% = 62$$

$$CE(5a) = \frac{29 \times 0.7}{7 \times 2.2 + 3 \times 1.1 + 13} \times 100 = 115$$

$$RME(5a) = \frac{449.7}{407.0 + 133.3 + 231.3} \times 100 = 58$$

$$OE(5a) = \frac{58}{88.9} \times 100 = 65$$

$$PMI(5a) = \frac{407.0 + 133.3 + 231.3 + 1578}{449.7} = 5$$

$$MP(5a) = \frac{1}{5} \times 100 = 20$$

$$EFactor(5a) = 20 - 1 = 19$$

$$SI(5a) = \frac{1578}{449.7} = 3.5$$

5.2 Two-step isolated process

Step 1: Synthesis of compound 3a

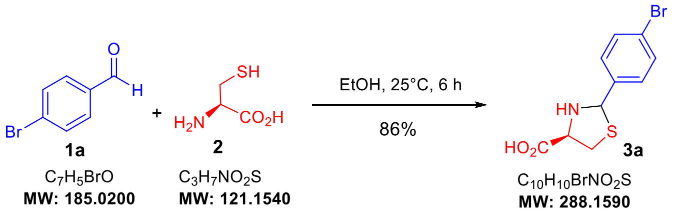

Experimental procedures:

To a solution of 4-bromobenzaldehyde **1a** (1.0 mmol) and in 10.0 mL of EtOH was added cysteine **2a** (1.15 mmol). After being stirred at 25 °C for 6 h. Upon the completion of the reaction as monitored by LC-MS, the reaction solution was slowly cooled down to 5-10 °C, then stirred for 3 h. The product **3a** was collected by filtration, after that, the residual filtrate was isolated on a semi prep-HPLC with C18 column (eluent, MeOH/H_2O = 60–75%) to afford the major product **3a** and combine two purified compound **3a** (86%).

Materials used for metrics calculations: 4-bromobenzaldehyde (**1a**, 185.0 mg, 1.0 mmol), cysteine (**2**, 139.3 mg, 1.15 mmol), EtOH (7890 mg, 10 mL) and compound **3a**, 247.8 mg (0.86 mmol).

$$AE(3a) = \frac{288.159}{185.02 + 121.15} \times 100 = 94$$

$$AEf(3a) = 94 \times 86\% = 81$$

$$CE(3a) = \frac{10 \times 0.86}{7 \times 1.0 + 3 \times 1.15} \times 100 = 82$$

$$RME(3a) = \frac{247.8}{185.0 + 139.3} \times 100 = 76$$

$$OE(3a) = \frac{76}{94} \times 100 = 81$$

$$MI(3a) = \frac{185 + 139.3 + 7890}{247.8} = 33$$

$$MP(3a) = \frac{1}{33} \times 100 = 3.0$$

$$E\ Factor(3a) = 3.0 - 1 = 2.0$$

$$SI(3a) = \frac{7890}{247.8} = 32$$

Step 2: Synthesis of product 5a

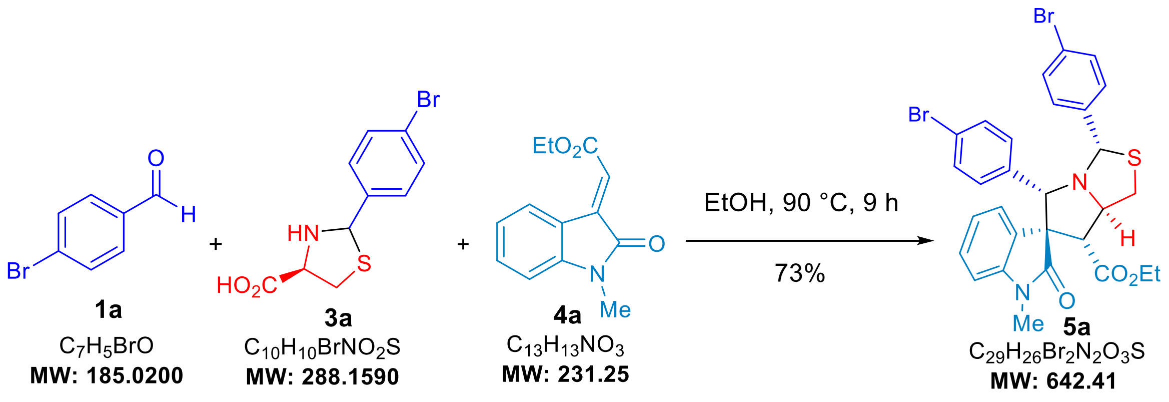

Experimental procedures:

To a solution of 4-bromobenzaldehyde **1a** (1.1 mmol) and intermediate **3a** (1.0 mmol), in 2.0 mL of EtOH was added olefinic oxindole **4a** (1.0 mmol). After being

stirred at 90 °C for 9 h. Upon the completion of the reaction as monitored by LC-MS, the concentrated reaction mixture was isolated on a semi-preparative HPLC with a C18 column (eluent, MeOH/H_2O = 70–90%). The major product **5a** was afforded (73%).

Materials used for metrics calculations: 4-bromobenzaldehyde **1a** (203.5 mg, 1.1 mmol), intermediate **3a** (288.2 mg, 1.0 mmol), olefinic oxindole **4a** (231.3 mg, 1.0 mmol), EtOH (1578 mg, 2 mL) and major product **5a** (469.0 mg, 0.73 mmol).

$$AE(5a) = \frac{642.41}{185.02 + 288.16 + 231.25} \times 100 = 91$$

$$AEf(5a) = 91 \times 73\% = 66.4$$

$$CE(5a) = \frac{29 \times 0.73}{7 \times 1.1 + 10 \times 1.0 + 13 \times 1.0} \times 100 = 69$$

$$RME(5a) = \frac{469.0}{288.2 + 203.5 + 231.3} \times 100 = 65$$

$$OE(5a) = \frac{65}{91} \times 100 = 71$$

$$MI(5a) = \frac{203.5. + 288.2 + 231.3 + 1578}{469.0} = 4.9$$

$$MP(5a) = \frac{1}{4.9} \times 100 = 20.4$$

$$E\ Factor(5a) = 20.4 - 1 = 19.4$$

$$SI(5a) = \frac{1578}{469.0} = 3.4$$

Cumulative metrics for compound 5a:

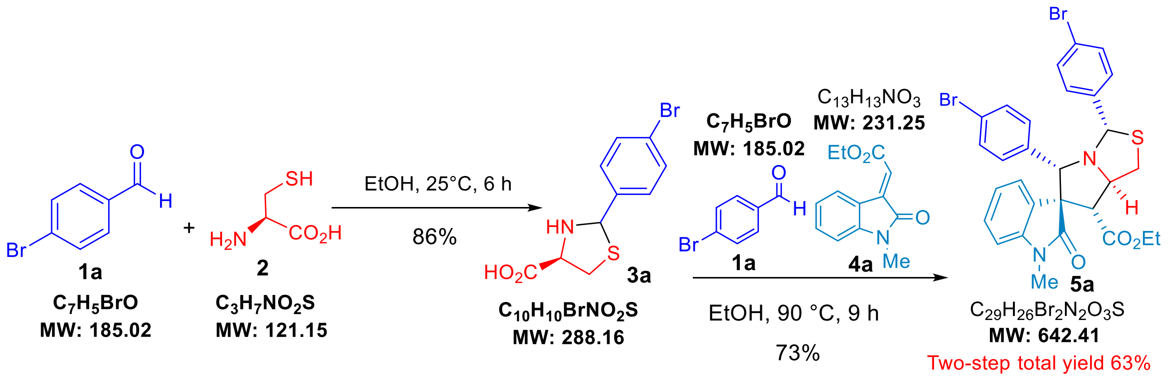

$$AE(5a\ \ cumulative) = \frac{642.41}{185.02 \times 2 + 121.15 + 231.25} \times 100 = 88.9$$

$$AEf(5a\,cumulative) = 88.9 \times 63\% = 56$$

$$CE(5a\ \ cumulative) = \frac{29 \times 0.73}{(7 \times 1.0 + 3 \times 1.15) \times \frac{1.0}{0.86} + 7 \times 1.1 + 13 \times 1.0} \times 100 = 64.4$$

$$RME(5a\ \ cumulative) = \frac{469.0}{\frac{288.2}{0.76} + 203.5 + 231.3} \times 100 = 57$$

$$OE(5a\,cumulative) = \frac{57}{88.9} \times 100 = 64.1$$

$$PMI(5a\,cumulative) = \frac{288.2 \times 33 + 203.5 + 231.3 + 1578}{469.0} = 25$$

$$MP(5a\,cumulative) = \frac{1}{25} \times 100 = 4$$

$$E\ Factor(5a\,cumulative) = 25 - 1 = 24$$

$$SI(5a\,cumulative) = \frac{7890 \times \frac{1.0}{0.86} + 1578}{469.0} = 23$$

6. References for green metrics

1. Trost, B. *Science* **1991**, *254* (5037), 1471-1477.

doi:10.1126/science.1962206

2. Roschangar, F.; Sheldon, R. A.; Senanayake, C. H. *Green Chem* **2015**, *17* (2), 752-768. doi:10.1039/C4GC01563K
3. McElroy, C. R.; Constantinou, A.; Jones, L. C.; Summerton, L.; Clark, J. H. *Green Chem* **2015**, *17* (5), 3111-3121. doi:10.1039/C5GC00340G
4. Willis, N. J.; Fisher, C. A.; Alder, C. M.; Harsanyi, A.; Shukla, L.; Adams, J. P.; Sandford, G. *Green Chem* **2016**, *18* (5), 1313-1318. doi:10.1039/C5GC02209F
5. Phan, T. V. T.; Gallardo, C.; Mane, J. *Green Chem* **2015**, *17* (5), 2846-2852. doi:10.1039/C4GC02169J
6. Abou-Shehada, S.; Mampuys, P.; Maes, B. U. W.; Clark, J. H.; Summerton, L. *Green Chem* **2017**, *19* (1), 249-258. doi:10.1039/C6GC01928E
7. Sheldon, R. A. *Green Chem* **2007**, *9* (12), 1273-1283. doi:10.1039/B713736M
8. Sheldon, R. A. *Green Chem* **2017**, *19* (1), 18-43. doi:10.1039/C6GC02157C
9. Jiménez-González, C.; Curzons, A. D.; Constable, D. J. C.; Overcash, M. R.; Cunningham, V. L. *Clean Products and Processes* **2001**, *3* (1), 35-41. doi:10.1007/PL00011310
10. Constable, D. J. C.; Curzons, A. D.; Cunningham, V. L. *Green Chem* **2002**, *4* (6), 521-527. doi:10.1039/B206169B
11. Wender, P. A.; Croatt, M. P.; Witulski, B. *Tetrahedron* **2006**, *62* (32), 7505-7511. doi:10.1016/j.tet.2006.02.085

12. Jimenez-Gonzalez, C.; Ponder, C. S.; Broxterman, Q. B.; Manley, J. B. *Org Process Res Dev* **2011**, *15* (4), 912-917. doi:10.1021/op200097d
